# A Combined CNN Architecture for Speech Emotion Recognition

**DOI:** 10.3390/s24175797

**Published:** 2024-09-06

**Authors:** Rolinson Begazo, Ana Aguilera, Irvin Dongo, Yudith Cardinale

**Affiliations:** 1 Electrical and Electronics Engineering Department, Universidad Católica San Pablo, Arequipa 04001, Peru; rolinson.begazo@ucsp.edu.pe (R.B.); ifdongo@ucsp.edu.pe (I.D.); 2 Escuela de Ingeniería Informática, Facultad de Ingeniería, Universidad de Valparaíso, Valparaíso 2340000, Chile; 3 Interdisciplinary Center for Biomedical Research and Health Engineering “MEDING”, Universidad de Valparaíso, Valparaíso 2340000, Chile; 4 ESTIA Institute of Technology, University Bordeaux, 64210 Bidart, France; 5 Grupo de Investigación en Ciencia de Datos, Universidad Internacional de Valencia, 46002 Valencia, Spain

**Keywords:** speech emotion recognition, deep learning, spectral features, spectrogram imaging, feature fusion, convolutional neural network

## Abstract

Emotion recognition through speech is a technique employed in various scenarios of Human–Computer Interaction (HCI). Existing approaches have achieved significant results; however, limitations persist, with the quantity and diversity of data being more notable when deep learning techniques are used. The lack of a standard in feature selection leads to continuous development and experimentation. Choosing and designing the appropriate network architecture constitutes another challenge. This study addresses the challenge of recognizing emotions in the human voice using deep learning techniques, proposing a comprehensive approach, and developing preprocessing and feature selection stages while constructing a dataset called EmoDSc as a result of combining several available databases. The synergy between spectral features and spectrogram images is investigated. Independently, the weighted accuracy obtained using only spectral features was 89%, while using only spectrogram images, the weighted accuracy reached 90%. These results, although surpassing previous research, highlight the strengths and limitations when operating in isolation. Based on this exploration, a neural network architecture composed of a CNN1D, a CNN2D, and an MLP that fuses spectral features and spectogram images is proposed. The model, supported by the unified dataset EmoDSc, demonstrates a remarkable accuracy of 96%.

## 1. Introduction

Human–Computer Interaction (HCI) has enabled the exchange of information between humans and machines, becoming a constantly evolving technology. Although HCI has been supported by touch screens and keyboards, speech remains an essential component of human culture, containing abundant data that can be processed for many proposals [1]. Speech recognition is a common practice for virtual assistants and robotic agents for many applications in everyday life that mostly require the user’s voice to operate. However, these applications are limited if they only consider basic aspects of speech, resulting in simple interactions without feedback [2].

To provide more effective HCI, speech recognition has been pushed a step further than just recognizing voice commands. By analyzing the rich data content of speech, it is possible to interpret and understand emotional information, which has shown to significantly improve HCI [3,4]. Therefore, research in this area has grown, exploring factors that enrich interaction, such as the user’s emotional state [5]. If a machine can interpret this information, it can respond in a way that influences human emotions, facilitating affective interaction [6].

Emotion recognition from speech allows devices to discern the user’s emotional state during interaction. Using this information, they can provide appropriate feedback, achieving affective HCI with great potential. For example, in medicine, the emotional state is an important information to determine patient well-being; in social networks, it can enhance interaction among users, as well as the response of voice assistants [2]; in music, musical emotion recognition aims to identify the affective tone of compositions, opening possibilities in areas such as personalized playlists, recommendation systems, and music therapy [7].

Existing approaches primarily rely on the use of artificial neural networks, traditional classifiers, and deep learning. The absence of a standard makes the choice entirely empirical [8]. However, the popularity of deep learning and artificial neural networks has increased due to the superior results obtained compared to traditional classifiers [9]. Moreover, these deep learning-based techniques entail higher development costs, such as the need for a vast amount of data for training [10]. Data collection, on the other hand, is costly and challenging, which limits the applicability of these classification techniques [11].

From the existing approaches and from our perspective, two of the most important aspects to approach in speech emotion recognition are the nature of the dataset used and the speech features considered during the preprocessing. There are various audio databases for emotion recognition, which are primarily classified into simulated, induced, and natural [8]. Choosing an appropriate database is crucial for the accuracy of real-world implementations. Although simulated and induced databases resemble real emotional states, they are not natural, which could lead to significant error rates in real-world applications [12]. Schuller et al. emphasize the importance of robust datasets and feature extraction methods in enhancing the accuracy and reliability of emotion recognition systems [13]. For this reason, natural databases have been developed, which provide information focused on real-world applications [14]. Regarding the preprocessing of the dataset, techniques such as silence removal and normalization are employed [15]. However, preprocessing audio signals might remove rich and relevant information [16].

In the current HCI scenario, discerning human emotions through voice emerges as a challenge that demands a rigorous and systematic approach. In this context, we propose a comprehensive approach composed of three development stages: preprocessing, feature selection, and classification. We have focused our efforts on a neural network architecture that integrates two Convolutional Neural Networks (CNNs), called CNN-1D and CNN-2D, and an MLP, employing two feature sources: spectral features and spectrogram images. This architectural and feature selection configuration aims to increase efficiency in terms of accuracy, mitigating the weaknesses that would arise from using the sources independently. In addition, we have formed a consolidated dataset, called EmoDSc. Recognizing the importance of data diversity, we have merged multiple databases to create a set that is distinguished not only by its richness of information but also by its utility for both training and testing.

In summary, our main contributions are:

- A comprehensive review of recent studies focusing on the development stages: preprocessing, feature selection, and classification. We provide a comparative evaluation of existing work in terms of the error metrics employed (such as accuracy), the classification method, and the databases used.
- A unified dataset, EmoDSc, which amalgamates several widely used databases, resulting in a diversified and information-rich database that can provide a varied and feature-rich training set.
- A neural network architecture that combines features, integrating a CNN-1D with a CNN-2D and culminating in an MLP. This meticulously designed structure not only enhances the recognition process but has proven its effectiveness by achieving a weighted accuracy of 96%.

We understand our work with a double vision of scalability: on the one hand, its practical implementation in social robots, with the aim of improving human–robot interaction (HRI)—an area in which we are also venturing—and, on the other hand, as a basis for future related work. Both the architecture and the integral development system can be improved and extended, either by going deeper into voice analysis or by venturing into multimodal recognition, integrating aspects such as facial gestures, textual analysis, and other components of human interaction.

We have structured the article as follows. In Section 2, we introduce the fundamentals of emotion recognition, emphasizing speech emotion recognition, and outline the typical process of developing a recognition system. In Section 3, we explore key research in the area, emphasizing the databases used, preprocessing techniques, feature selection, and predominant classification techniques, along with their respective performance. A detailed description of our methodological proposal, outlining each step from database selection to the construction and fine-tuning of the neural network architecture, is presented in Section 4. In Section 5, we present an exhaustive discussion of our results. We evaluate the feature sources individually before combining them and we test the architecture with additional databases to measure its robustness and versatility. Deep reflection on the findings and progress made, identifying strengths, limitations, and opportunities for improvement, is presented in Section 6. Finally, in Section 7, we point out conclusions and future research in this exciting field.

## 2. Emotion Recognition: Preliminaries

For the correct implementation of a speech emotion recognition system, it is necessary to define what emotions are. While there is no standard definition, it can be understood that emotions are psychological states which are composed of physiological, behavioral, and communicative reactions [12].

Emotion recognition is a diverse discipline with various approaches that includes identifying emotions through several modalities. Facial and body gestures, for example, can be interpreted through images [17], while textual analysis extracts semantic features to determine emotional states [18]. Physiological signals have also gained recent attention as an emotional detection tool [19]. Each technique has its advantages and disadvantages, and the choice usually depends on the data acquisition modality [17].

Due to their intrinsic nature in human communication, voice and speech stand out in this context for their simplicity and effectiveness [17]. The variety of applications is vast and depends on the data source used, whether it is voice, gestures, or even a combination of these, leading to multimodal recognition. This last option, despite its complexity, has shown promising results in various research [18].

In the context of speech emotion recognition, it is vital to differentiate between “voice” and “speech”. Although these terms are often used interchangeably, as shown by Alu et al. [20], they have distinct connotations. The “voice” specifically refers to the sound produced by the vocal cords that is propagated through the vocal apparatus. On the other hand, “speech” has a broader scope, encompassing not only the voice but also the articulation of words and phrases; it is perceived as a natural and primary form of human communication [12]. Thus, “speech emotion recognition” goes beyond just examining linguistic content. It can identify emotions based on various speech features, whether linguistic content, prosodic aspects like tone or intonation, or spectral characteristics.

Today, a speech emotion recognition system is divided into three stages: speech signal preprocessing, feature extraction, and classification; moreover, the second stage is the most important as it is the main source of descriptors for the model [21]. Likewise, deep learning-based techniques have become popular in stages such as classification and feature extraction. These techniques, a subset of machine learning, are widely used in data science and analysis for their ability to learn from large volumes of information [22].

**Figure 1:**
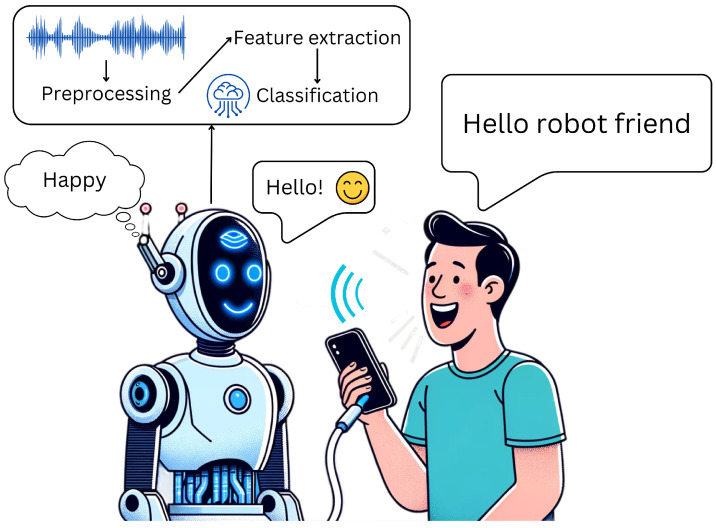
Application example of an emotion recognition system.

The process begins with data preparation, selecting an appropriate dataset for training. Commonly, these datasets are generated through stakeholder participation. In relation to the development stages, the usual first step is preprocessing. This stage is implemented for the purpose of preparing the data for feature extraction, normalizing the information to compensate for variations present in the audio [12]. Feature extraction is a crucial aspect in speech emotion recognition. Generally, a set of features is elaborated based on empirical approaches [12]. Regarding classifiers, one can opt for traditional classifiers or deep learning algorithms. Figure 1 illustrates in a simple way the typical process at the application level in speech emotion recognition.

## 3. Related Work

In the field of HCI, numerous studies have addressed the challenge of emotion recognition in speech. These works have focused on different stages of the process, from data acquisition to the implementation of advanced analysis techniques. In this section, each of these stages are reviewed, understanding the applied techniques, methodologies, and results.

### 3.1. Datasets for Speech Emotion Recognition

Research aimed at improving HCI through speech emotion recognition has proliferated in recent years. Within this scope, the application of machine learning techniques, and particularly deep learning, has stood out for its ability to model and classify complex patterns. It is essential to emphasize that the success of these techniques largely depends on the quality and quantity of available data [1]. Not all datasets are the same: they vary in the number of emotions, language, and accessibility, among other factors [14]. It is known that the accuracy of a system can vary significantly depending on the language and the number of emotions considered, further complicating systems that try to identify a broader emotional range [16].

In the landscape of databases dedicated to emotional recognition, it is common to find categorizations that include emotions, such as “anger”, “disgust”, “fear”, “happiness”, “sadness”, and “surprise”. These categories largely align with the proposal of basic emotions by Ekman [23]. While this set of emotions has been established as a standard in many databases, some may incorporate additional categories. Regarding access, databases vary in terms of availability. Some are offered for free and are easily accessible online. Others, especially those intended for academic purposes, may require contacting the authors or related institutions to request a license, usually justifying their use in specific research, while some databases operate under paid schemes, reflecting the investment and value that these specialized resources represent.

Regarding deep learning techniques, choosing a robust database is essential as it is the main pillar for training, especially for deeper architectures [24]. A larger and more diverse database can allow identifying more relevant features, improving classification effectiveness. While English predominates in many datasets, as reflected in Table 1, it is crucial to consider factors such as language and database accessibility when choosing the appropriate set for research.

There is a consensus in various studies on the categories of databases used for emotion classification, where the vast majority agree that there are simulated, induced, and natural ones. Simulated databases are generated from performances by experienced actors [8]. Induced ones are derived from situations designed to evoke emotions in uninformed individuals, resulting in emotional expressions that are closer to real ones [8]. On the other hand, natural ones, also known as in-the-wild, often recorded in public environments, offer a more realistic and authentic representation of emotions [8].

Recent efforts, such as the Odyssey 2024 Speech Emotion Recognition Challenge, emphasize the use of natural databases like MSP-Podcast, which capture spontaneous emotional expressions in real-world contexts [25]. These datasets are more reflective of everyday interactions, offering a valuable benchmark for developing robust emotion recognition systems. However, while natural databases are highly valued for their spontaneity, they present significant challenges due to the variability of emotions and environmental noise, making emotion recognition in these contexts more complex.

**Table 1:** Popular Databases.

Database	Emotions	Language	Category	Access
RAVDESS dataset [26] (2018)	Anger, Disgust, Fear, Happiness, Neutral, Calm, Surprise, Sadness. (1440 audio files)	English	Simulated	Free
RAVDESS SONG [26] (2018)	Calmness, Happiness, Sadness, Anger, Fear, Surprise, Disgust. (1012 audio files)	English	Simulated	Free
MSP-Podcast [27] (2019)	Anger, Happiness, Sadness, Neutral, Disgust. (62,140 speaking turns, 100 h)	English	Natural	Academic request
SAVEE [28] (2009)	Anger, Disgust, Fear, Happiness, Sadness, Surprise, Neutral. (480 audio files)	English	Simulated	Free
Crema-D dataset [29] (2014)	Anger, Disgust, Fear, Happiness, Sadness, Neutral. (7442 audio files)	English	Simulated	Free
JL Corpus [30] (2018)	Anger, Sadness, Neutral, Happiness, Excitement, Anxiety, Thoughtfulness, Enthusiasm, Worry (1080 audio files)	English	Simulated	Free
IEMOCAP [31] (2008)	Anger, Happiness, Neutral, Disgust, Fear, Sadness, Excited. (12 h of audiovisual data, including 10,039 utterances)	English	Induced	Academic request
Keio-ESD [32] (2006)	Anger, Happiness, Funny, Downgrading, Disgust, Worried, Gentle, Relief, Indignation, Shameful. (A set of human speech with vocal emotion spoken by a Japanese male speaker.)	Japanese	Simulated	Academic request
ASVP-ESD [33] (2021)	Boredom, Neutral, Fear, Sadness, Happiness, Anger, Surprise, Disgust, Excitement, Pleasure, Pain, Disappointment. (13,285 audio files collected)	Chinese, English, French, Russian	Natural	Free
CASIA [34] (2003)	Happiness, Sadness, Anger, Surprise, Fear, Neutral. (1200 audio files)	Chinese	Simulated	Free
EMODB [35] (2005)	Anger, Fear, Disgust, Sadness, Happiness, Boredom, Neutral. (535 audio files)	German	Simulated	Academic request
ESD [36] (2021)	Anger, Happiness, Neutral, Sadness, Surprise. (29 h, 3500 sentences)	English	Simulated	Free
SUSAS [37] (1997)	Anger, Fear, Surprise, Neutral, Sadness, Happiness. (16,000 audio files)	English	Simulated and Natural	By purchase
AFEW5.0 [38] (2014–2015)	Anger, Disgust, Fear, Joy, Neutral, Sadness, Surprise. (1809 audio files)	English	Natural	Academic request
BAUM-1s [39] (2018)	Anger, Disgust, Fear, Joy, Sadness, Surprise. (300 audio files)	English	Simulated and Natural	Academic request
TESS [40] (2010)	Anger, Boredom, Fear, Happiness, Neutral, Sadness. (2800 audio files)	English	Simulated	Free

Although natural databases are highly valued for their spontaneity [1], they present significant challenges. For example, due to the fluidity with which emotions change in real situations and environmental noise, determining a specific emotion can be complicated [24]. However, the ability to recognize emotions is crucial in different environments [14], so a natural database is ideal for this purpose. Table 1 presents a summary of the most popular databases in the reviewed literature, some of which are used in this work.

### 3.2. Preprocessing Techniques Applied to Audio Signals

There are various techniques or methods applicable to the preprocessing stage in an emotion recognition system; however, some studies omit this stage. One reason could be that preprocessing can remove valuable information from the dataset [16], making it preferable to use the raw signal for subsequent stages such as feature selection. This trend of not applying preprocessing is also common when the main source of information is spectrogram images [2,41].

Other studies that extract traditional features or apply a specific feature selection also dispense with preprocessing [42]. Nevertheless, this does not imply that preprocessing is unnecessary. In fact, popular techniques such as silence removal aim to remove irrelevant information and retain only speech [15,43,44]. Some works choose to remove only the silent parts of the ends [9]. In addition to this technique, there are methods to remove unwanted noises [43].

Normalization is also common, present in the studies of Lee and Kim [45], Harár et al. [15], Singh et al. [46], Mishra et al. [47], Deshmukh et al. [44], and Pappagari et al. [11]. Likewise, some studies use data augmentation with different techniques, such as Abdelhamid et al. [10] and Singh et al. [46] that add noise, and Pappagari et al. [11], who concatenate audio. In terms of normalization, in the context of spectrogram images, scaling is often applied [48]. Along with these images, segmentation [48,49] and windowing [50] are also common. Also, silence removal and noise reduction techniques were employed to preprocess the audio signals, in addition to applying Z-normalization to standardize the features across the entire dataset [51].

There are a variety of preprocessing techniques, some more prevalent than others. Although it is not always the central stage, there are studies that apply multiple techniques together, such as sampling, quantification, pre-emphasis, framing, window addition, and endpoint detection [21,52].

Moreover, it is worth mentioning techniques like the study presented by y Mustaqeem et al. [49], which divides the speech signal into segments and then regroups them by K-means clustering, resulting in a sequence of key segments, to which the Short-Time Fourier Transform (STFT) is applied to obtain spectrograms. In summary, although there are several preprocessing techniques, their choice depends on the proposal and the features to be used. It is essential to take into account this stage, since the response speed of a system may depend on the complexity of each phase.

### 3.3. Feature Selection

This stage is also known as “feature extraction”, but to give a broader focus, we call it “feature selection”, as the source of information for classification can come from a manual selection of traditional features, such as spectral, prosodic, and voice quality [1]. However, it is also possible to use spectrogram and mel-spectrogram images [48] or directly employ neural network architectures for feature extraction [21]. Various traditional features can be classified. Although this may vary depending on the work, three categories are generally considered: prosodic, spectral, and speech quality [12]. Additionally, there are studies that use hyper-prosodic features, which have been termed statistical features [41].

Although there is no particular set of universally accepted features, the most commonly used are traditional ones (i.e., those extracted directly from the speech signal, such as spectral or prosodic). However, there is no predetermined set or selection that guarantees success in predicting emotions in speech [12]. Most studies resort to experimentation to select the most suitable features. This has given rise to recognized feature sets, such as the ComParE openSMILE, which allows extracting a tested set. In the work developed by Xie et al. [53], this dataset is used along with a subset called Frame-Level features, using two classifiers for each feature source.

Studies such as those proposed by Lee et al. [9], Pappagari et al. [11], Harár et al. [15], and Issa et al. [54] employ varieties of traditional features, mainly spectral and prosodic. It is also possible to combine sources, using traditional features and other resources. For example, Lee and Kim [45] use spectral features and also mel-spectrogram images, while Liu et al. [41] use statistical and hyper-prosodic features along with spectrogram images. There are also multimodal studies; Cai et al. [2] combine spectral features with facial gestures and Yoon et al. [55] add text to their set of spectral and prosodic features.

Other studies take a more comprehensive approach towards multimodal recognition, as shown in the work of work of Graterol et al. [56] and Heredia et al. [57], where different sources of information such as speech, image, and text are incorporated. These data sources provide a wealth of information that enhances prediction accuracy; however, from an application perspective, it requires the availability of multiple high-quality sensors. In addition, it requires the availability of extensive datasets for image (either facial or body modality), voice and text; and it is crucial that the training data be representative of the environment in which the solution is planned to be deployed [56].

Selecting relevant features is a challenge. Aouani and Ayed [42] add a broad set of spectral and prosodic features but finalize their selection with an AutoEncoder to determine the final parameters. On the other hand, traditional features are not mandatory; studies such as those presented by Zhang et al. [48], Mustaqeem and Kwon [43], Li et al. [50], Wu et al. [58], and Abdelhamid et al. [10] rely on one of two options, spectrogram or mel-spectrogram images.

Neural networks are also popular for feature extraction. Zhao et al. [59] implement deep learning with local feature learning blocks (LFLBs) and Long Short-Term Memory (LSTM), also adding artisanal features. Additionally, a Deep Belief Network (DBN) can be used for feature acquisition, as in the research by Wang and Han [52] and Han and Wang [21]. Finally, transfer learning is another option for extraction, using known architectures such as Residual Network (ResNet) [49]. Acoustic features extracted from mel-spectrograms are also utilized, where Z-normalization is applied [60]. Other studies present feature extraction methods that combine MFCCs with spectral entropy and approximate entropy to extract meaningful features from segmented audio fragments [47].

Several studies have demonstrated the effectiveness of using spectral features and spectrogram images for emotion recognition. Lee and Kim [45] successfully utilized a combination of spectral features and mel-spectrogram images to enhance emotion classification performance. Similarly, Liu et al. [41] integrated statistical and hyper-prosodic features with spectrogram images, showing significant improvements in accuracy. Zhang et al. [48], Mustaqeem and Kwon [43], and Wu et al. [58] also relied solely on spectrogram or mel-spectrogram images, demonstrating their robustness and ability to capture essential emotional cues in speech. These studies support the viability of using spectral features and spectrogram images, aligning with our approach to leverage these techniques for better performance in emotion recognition tasks.

### 3.4. Classification Techniques

In the development of a speech emotion recognition system, the need arises to determine which type of classifier is most suitable. Commonly, machine learning based techniques prevail, but within this broad field, deep learning has gained notable prominence [24]. This trend is justified by its ability to handle unstructured data, making it especially convenient for handling information derived from voice, images, and text [61]. In the vastness of scientific literature, it is possible to identify a variety of methods, techniques, and algorithms. Each study proposes different approaches, many of which are forged empirically and through experimentation [12].

In the study proposed by Lee et al. [9], a Support Vector Machine (SVM) and a Deep Neural Network (DNN) are used. When contrasting both methods with an identical set of features, the DNN excelled. However, Aouani and Ayed [42], by exclusively implementing an SVM, manage to surpass even the DNN of Lee et al. [9]. This indicates that the choice of classifier does not determine the success of a system, but the quality of preprocessing and feature selection are equally crucial.

On the other hand, the study proposed by Han and Wang [21], which employs Proximal SVM (PSVM), achieves an error rate of only 5%. This result is particularly notable, considering that neural networks are not used. The success of this work lies in its meticulous preprocessing phase and in the feature extraction that incorporates a DBN.

Neural network architectures like ResNet have proven to be promising and versatile. Pappagari et al. [11], for example, use ResNet as a classifier, obtaining F1-score results below 78%. Despite this, their research measures the performance of large neural networks in speech emotion recognition.

Various studies have proposed the use of architectures such as DNNs, multilayer perceptrons (MLPs), and CNNs. For example, Lee and Kim [45] implement an MLP and a CNN with favorable results for the MLP; Liu et al. [41] and Cai et al. [2] explore the capabilities of CNNs and DNNs, obtaining diverse results based on the features and methods used.

Beyond standard classifiers, CNNs have been adapted to achieve greater sophistication. Zhang et al. [48] and Meng et al. [62], for example, explore multi-CNN variants, while Sun [16] experiments with a Residual CNN (R-CNN). LSTM architectures, interesting proposals used by Abdelhamid et al. [10] and Zhao et al. [59], have proven to be particularly efficient, showing exceptional accuracies. Additional classification techniques include the use of Temporal Convolutional Networks and attention-based models, demonstrating their effectiveness in specific scenarios [46].

Finally, there are techniques less prevalent in the literature, but no less promising, such as the Shallow Neural Network [52], the Capsule Networks [58], and the Probabilistic Neural Network (PNN) [44]. The PNN, in particular, has demonstrated high accuracy in speech emotion recognition, achieving 95.76% accuracy on the EMO-DB dataset and 84.64% on the RAVDESS dataset. There is no single technique that guarantees success in emotion recognition, allowing for a continuous flow of innovation. This constant evolution in the field is not only inevitable but also enriching for future research in the area.

### 3.5. Review of Techniques and Methods

The literature review has addressed a wide range of works, emphasizing the different techniques implemented in the essential stages for the construction of an emotion recognition system, from preprocessing to feature selection or extraction, culminating in the classification phase. This complete overview is detailed in Table 2 and Table 3, which synthesize key data from each analyzed research. Based on the collected information, it is evident that, even when working with the same database, an identical feature selection, or the same classification algorithm, the predictive performance of a speech emotion recognition system is a result of the sum of its parts. Each stage has a significant impact on the final product. This inherent uniqueness to each study, where the stages do not produce uniform results, highlights that development is trial and error, that is, experimentation.

## 4. Speech Emotion Recognition Based on a Combined CNN: Our Proposal

**Figure 2:**
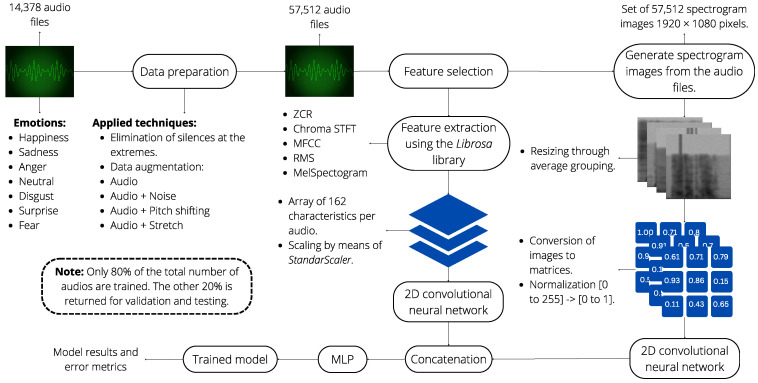
Proposal flow.

Figure 2 provides an overview of our proposed workflow for speech emotion recognition. The flow is segmented into three phases: (i) data preparation; (ii) feature selection; and (iii) classification. The following sections describe each phase of our proposal.

### 4.1. Data Preparation: EmoDSc Dataset Creation

In this work, we favor the use of raw audio and the combination of multiple databases to obtain a diverse and information-rich dataset. Additionally, there are techniques like data augmentation that improve model generalization [63]. It is essential to have an optimal feature selection to train a deep learning model. Generally, feature selection is based on experimentation and there is no set that ensures success in predictions [1]. Something similar happens with classification; there are many techniques and methods, so many deep learning based algorithms are developed empirically [12].

#### Combination of Databases

In this study, we emphasize the identification of a variety of emotions. Due to the considerable volume and variety of data required, we chose to combine multiple databases to expand and enrich the information linked to each emotion. These databases contain data of diverse characteristics, such as unique attributes, distinct duration, and varied recording qualities. While this heterogeneity could present challenges in terms of consistency and accuracy, it is precisely the diversity that we seek to address in this work. The goal is to train the model with a broad spectrum of information, mimicking conditions that would be encountered in real situations. We consider the following characteristics when evaluating a database:

- **Emotions**: The choice of emotion sets is diverse and is not usually deeply addressed in many studies. This selection is often linked to the database used, as not all databases present the same emotional categories. In this study, we decided to work with databases that include the following emotions: “happiness”, “sadness”, “anger”, “disgust”, “surprise”, “fear”, and “neutral”.
- **Language**: Language plays a crucial role due to variations in pronunciation, tone, and speech rhythm among different languages. Although there are multiple languages, English, often considered the universal language, predominates in the most popular databases. This wide availability of English datasets is the reason it was selected as the primary criterion for this study.
- **Category**: Although it has been previously mentioned that databases can be categorized as simulated, induced, or natural, based on their origin, in this study, such categorization will not be determinant in the selection. Instead of focusing on the specific nature of the database, we prioritize the diversity of the information it contains.

For the selection of the datasets, eight databases have been chosen according to the previously established criteria. The selected databases include both simulated and natural emotion data to ensure a comprehensive evaluation of the emotion recognition system across different types of data. The databases chosen are:

- **RAVDESS and RAVDESS SONG:** These databases contain simulated emotional expressions with a variety of emotions, including anger, disgust, fear, happiness, neutral, calm, surprise, and sadness. The simulations are performed by actors, providing a controlled environment ideal for testing model robustness with clear, distinct emotional expressions [26].
- **SAVEE:** This database also features simulated emotions, focusing on basic emotions like anger, disgust, fear, happiness, sadness, surprise, and neutral. The controlled nature of this dataset makes it suitable for testing fundamental aspects of the emotion recognition models [28].
- **Crema-D:** Another simulated emotion dataset, Crema-D offers a broader range of vocal expressions from a diverse group of actors. This diversity in speakers and controlled simulations helps in assessing the model’s generalization capabilities across different voices and expressions [29].
- **TESS:** The TESS dataset includes simulated emotions expressed by older female speakers, providing a unique angle on how age and gender might affect emotion recognition in speech [40].
- **JL-Corpus:** Similar to the other simulated datasets, JL-Corpus includes a wide range of emotions but focuses on expressions that may be more nuanced, adding complexity to the emotion recognition task [30].
- **ASVP-ESD:** Unlike the previous datasets, ASVP-ESD includes natural emotions, recorded in real-world environments, which introduces variability and noise that better reflect real-life scenarios. This makes it a crucial addition to the dataset selection, allowing the model to be tested on more spontaneous and less controlled emotional expressions [33].
- **ESD:** This dataset contains a large number of sentences spoken in English with simulated emotions, offering extensive training material and contributing to the overall diversity of the dataset collection [36].

While most of the selected databases are simulated, reflecting the majority of controlled emotion recognition research, the inclusion of ASVP-ESD, a natural emotion dataset, addresses concerns about the applicability of the models to real-world scenarios. The combination of these datasets ensures that the models are robustly trained and evaluated across a spectrum of emotional expressions, from controlled simulations to more spontaneous, natural occurrences, providing a balance between experimental rigor and practical relevance.

Besides contemplating the seven distinct emotions specified before, we consider the emotions “calm” and “neutral” as equivalent. The emotion “neutral” is not strictly an emotion per se, as it does not significantly affect the individual’s behavior or thinking. Rather, it represents a state of tranquility. For this reason, in our study, both emotions are treated as a single category.

Since not all of these selected databases include the complete set of emotions previously defined, it was necessary to select specific segments from each database. First, we filtered each database to select only the emotions required for our study, using the labels provided by the original authors. This process resulted in a large but unbalanced dataset. To balance the dataset, we then performed a proportional random selection from each database, ensuring that each emotion was equally represented. After this process, our resulting database, that we named EmoDSc, accumulated a total of 14,378 voice files, 2054 audio files for each emotion. Thus, we have a balanced dataset.

### 4.2. Preprocessing

Although we are not constrained by specific hardware, it is prudent not to overload the preprocessing phase with unnecessary steps, as this can affect overall system response times. Proper preprocessing can also speed up training, for example, by eliminating silences at the beginning and end of recordings. Studies such as those by Mustaqeem et al. [49] have shown that focusing on key segments of audio can significantly reduce processing time and computational load, making the system more efficient and suitable for real-time applications. Additionally, normalizing CNN features has been demonstrated to stabilize training and improve recognition performance without adding significant computational overhead. Therefore, the various techniques applied during this preprocessing phase of our EmoDSc are carefully chosen to balance computational efficiency and accuracy. The preprocessing steps applied are as follows.

#### 4.2.1. Data Augmentation

It is a strategy frequently employed in model training to generate a larger and more diverse dataset. Its objective is to present the model with a variety of scenarios and conditions, thus facilitating its adaptation to complex situations during the prediction phase. However, this augmentation process is limited to the development period and focuses on enriching the training set. Since deep learning models are capable of learning massive amounts of data [64], this data augmentation stage is useful.

In real-world scenarios, several challenges arise, such as the presence of noise, possible errors in speech recording or transmission, and even variations in pitch, which may depend on the device used for recording. While it would be ideal to have high quality devices that guarantee clean recordings, the reality can be the opposite. In this context, while our goal is to train the model with clear data, it is ideal that the model also be robust to these common adversities. Therefore, part of our preprocessing is aimed at giving the data a more “realistic” character, simulating the conditions that the model might encounter in a possible practical application.

**Figure 3:**
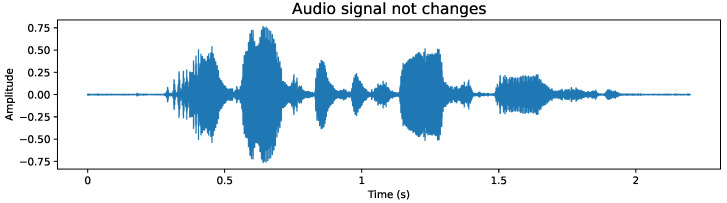
Base audio.

The audio files have a form like the example in Figure 3, with no added modifications or disturbances.

**Figure 4:**
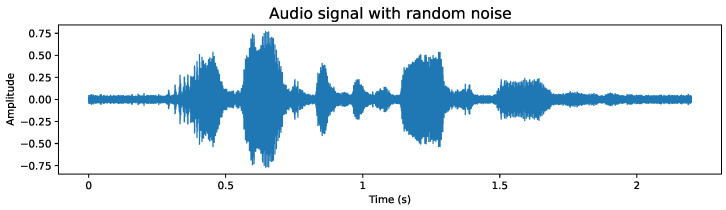
Added noise.

**Figure 5:**
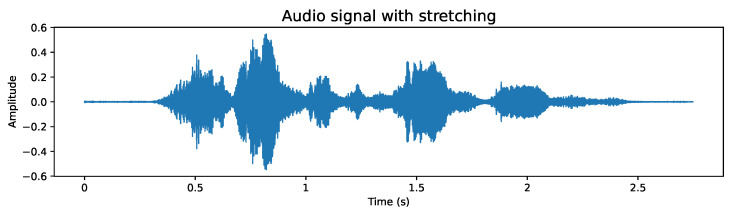
Stretching.

**Figure 6:**
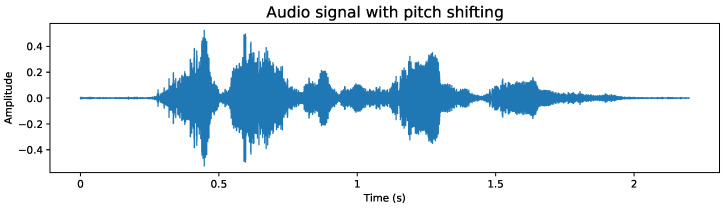
Pitch shifting.

To apply this data augmentation, we generated audio files that incorporate the following modifications:

- **Add noise:** Given that in real environments it is unlikely to always have a clear voice signal, it is essential to include noise in our recordings to simulate these conditions and adequately prepare the model. Although the white noise we add is random and not directly related to the original signal, this type of alteration helps approaching a more realistic scenario. The addition of noise can be exemplified in Figure 4, where disturbance can be observed throughout the audio signal, a feature not observed in Figure 3.
- **Stretching:** This technique modifies the duration and speed of the audio, simulating situations where the recording has not been made under ideal conditions. By including audios that have been stretched or compressed in time, the model is prepared to adapt and recognize patterns in recordings that may not have a constant speed, thus strengthening its ability to face real challenges. Figure 5 exemplifies this modification, where it can be seen that the duration of the audio increased compared to the base audio in Figure 3 due to the applied stretching.
- **Pitch shifting:** The pitch of a recording may vary depending on the quality of the microphone used. It can even vary if the person is constantly moving closer and further away in their interaction. To ensure that the model is robust to these variations and can operate in less than ideal conditions, we introduce intentional changes to the pitch of the recordings. This creates a more diverse and demanding dataset, which trains the model to cope with recordings of varying quality. Figure 6 shows these modifications; compared to Figure 3, a similar signal shape is obtained, but with clear differences in amplitude in some segments.

After applying the augmentation techniques, we have an expanded EmoDSc, with the total amount to 57,512 audio files, distributing 8216 voice recordings equally for each emotion.

#### 4.2.2. Removal of Silences at the Ends

**Figure 7:**
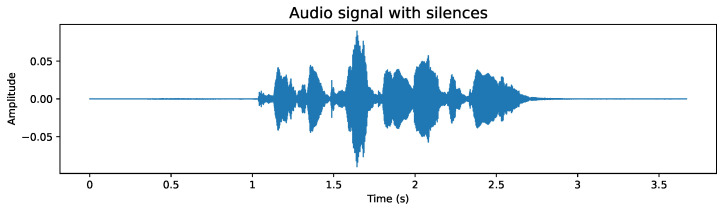
Voice signal with silences at the ends.

**Figure 8:**
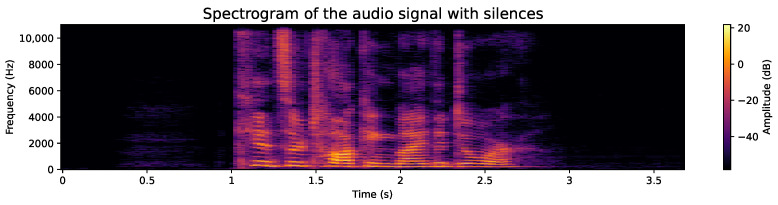
Spectrogram of the voice signal with silences at the ends.

**Figure 9:**
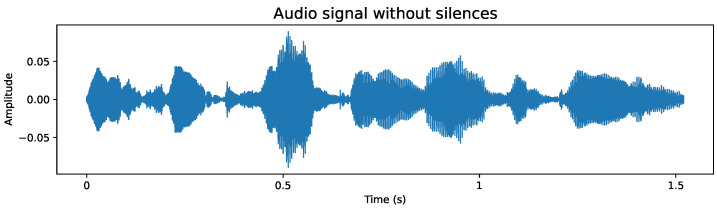
Voice signal without silences at the ends.

**Figure 10:**
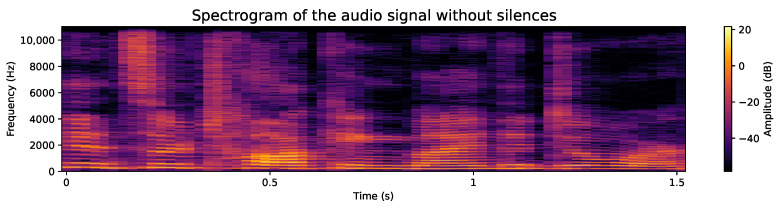
Spectrogram of the voice signal without silences at the ends.

While the use of the raw audio signal is encouraged, the elimination of silences plays a beneficial role in development efficiency, as the processing of shorter audios significantly reduces the time required. In addition, the silent content at the ends could be considered irrelevant as it is not part of speech. Example of samples from the database, before and after removal of silences, are as follows:

- **Original audio sample**: Figure 7 shows an example of an audio file whose duration exceeds 3.5 s. Although any audio with its original length could be used, this would mean an increase in the use of computational resources and an extension of processing time, both in model training and feature extraction. Additionally, the silences detected at the end of the audio do not provide essential speech information. This premise is corroborated when analyzing the spectrogram, such as the one shown in Figure 8, where areas lacking energy at their ends are evident, indicating the absence of relevant information. Therefore, it is appropriate to dispense with these silent segments.
- **Audio sample after silence removal**: The entire database has been processed to remove the silences at the beginning and end of each audio. An example of this is shown in Figure 9, which is the processed version of the audio presented in Figure 7. It is notable that the audio duration has been reduced to just over 1.5 s. Although the reduction in a single file may seem insignificant, when considering the complete set of over 50,000 voice files, the cumulative decrease in processing time is considerable, translating into significant computational resource efficiency. Likewise, the spectrogram presented in Figure 10 shows a higher information density, with regions of higher energy, indicating more concentrated and relevant content for subsequent analysis.

#### 4.2.3. Creation and Use of Spectrogram Images

The spectrogram, as previously mentioned, is an invaluable tool for analyzing energy in the voice signal, providing deep features about the emotions that may be present in that signal. While humans can naturally decipher and understand emotions just by listening to a tone of voice, for a machine, this task is much more complex. The system does not “listen” to the voice in the human sense; instead, it analyzes the information contained in that voice signal.

Several works, such as those proposed by Lee and Kim [45] and Liu et al. [41], have adopted spectrograms as a data source for training neural networks. Although it is not a novelty per se, it is essential to implement appropriate techniques that allow the effective use of these spectrogram images. CNNs are particularly suitable for this type of task, as they are primarily designed for image classification.

With the removal of silences from the audio files, it is possible to generate high-quality spectrogram images. However, although the audio recordings have different durations, which might suggest that the spectrograms would vary in size, this does not affect the frequency or amplitude dimensions of the spectrogram. What actually happens is that the energy is distributed differently in the spectrogram according to the duration of the audio, e.g., longer recordings tend to have the energy more “compressed” along the temporal axis. This phenomenon can be challenging, as temporal compression can cause visual representations to differ in terms of energy dispersion. However, we have chosen to generate the spectrograms with uniform dimensions, maintaining consistent time and frequency scales across all images. Although this means that some images will show energy more concentrated in time than others, we consider this variability to be valuable. It reflects the diversity of real-world scenarios where audio durations can vary significantly, and it is crucial that the model is prepared to handle different temporal scenarios.

**Figure 11:**
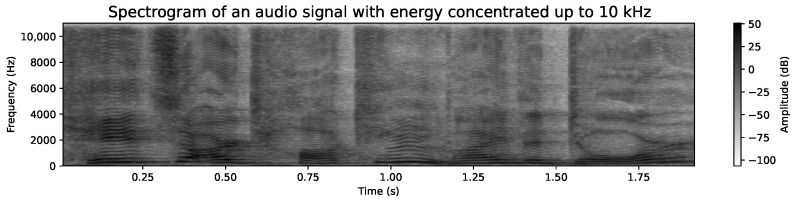
Spectrogram of an audio signal containing significant energy up to 10 kHz.

**Figure 12:**
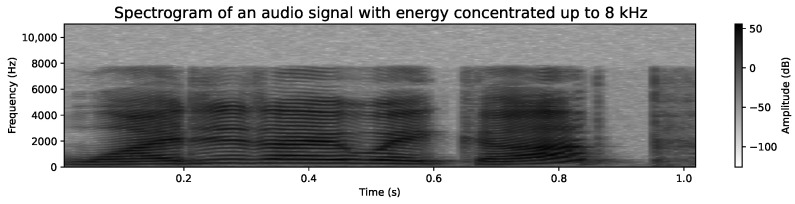
Spectrogram of an audio signal where most of the energy is concentrated below 8 kHz.

Additionally, we have opted to work with grayscale images. Each pixel in the spectrogram reflects the signal intensity in decibels (dB), and the use of color is simply a convention to facilitate visualization. In our case, grayscale representation reduces computational complexity by eliminating the need to handle multiple color channels, which is particularly beneficial for processing efficiency in neural networks. This allows the focus to remain on essential frequency information without compromising the quality of the analysis. Figure 11 and Figure 12 show the grayscale representations, which are coherent and clear, maintaining the integrity of the voice signal analysis.

Regarding the frequency range, we have used an upper limit of 10 kHz. Although some studies, such as Meng et al. [62], opt for a range of 300 Hz to 8000 Hz, we believe that analyzing up to 10 kHz can provide additional relevant information for emotion detection. While most of the energy in the human voice is concentrated below 8 kHz, components such as higher harmonics, which can extend up to 10 kHz, might contain subtle cues related to emotion, clarity, or voice tone. These details, although less prominent, could improve the model’s accuracy by providing a more complete representation of the voice signal. It is important to note that not all audio files contain significant information above 8 kHz, but for those that do, these higher frequencies can offer valuable additional context for emotional classification. To illustrate this variability, Figure 11 shows a spectrogram with significant energy up to 10 kHz, while Figure 12 shows another spectrogram where the energy is mostly concentrated below 8 kHz. This diversity in spectral representation is expected and underscores the need to manage a broad range to capture all possible nuances in the voice signal.

- **Resizing of spectrogram images**:

Dimensionality reduction is a crucial step in image processing for training machine learning systems, especially neural networks. This practice can significantly accelerate training time and reduce resource consumption, provided that it is performed in a way that preserves essential information.

**Figure 13:**
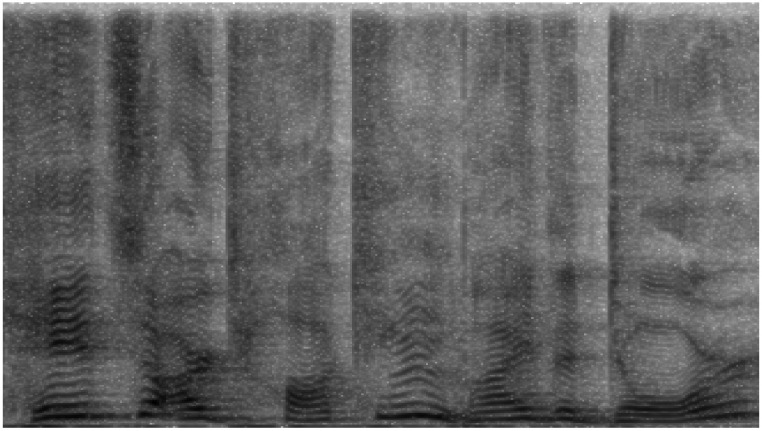
Spectrogram of the audio signal without silences (grayscale, 1920 × 1080 pixels).

**Figure 14:**
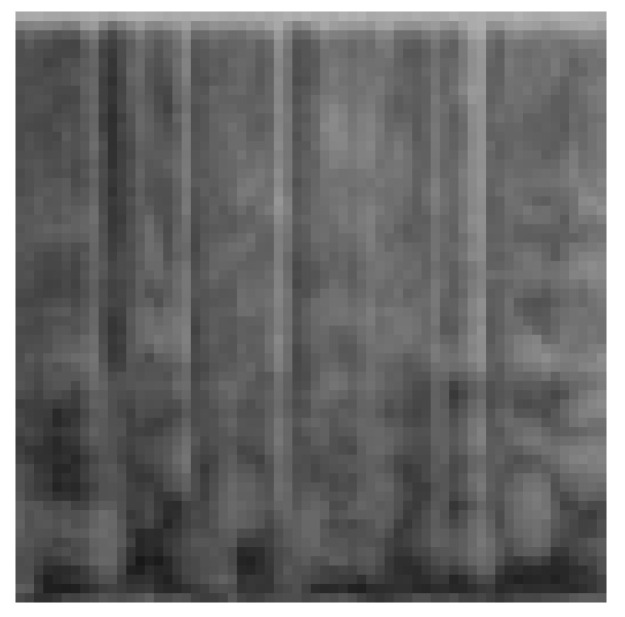
Spectrogram of the audio signal without silences (grayscale, 64 × 64 pixels).

Initially, spectrograms were generated in high resolution (1920 × 1080 pixels) to capture the maximum level of detail possible in the audio signals (Figure 13). This approach is based on the idea that higher resolution allows for the preservation of important signal characteristics, such as subtle patterns and transients, which might be lost at lower resolutions. Subsequently, these images were reduced to a more manageable resolution of 64 × 64 pixels using the average pooling technique, which aims to retain the general structure of the image at a lower resolution (Figure 14).

Although we recognize that double conversion (from 1920 × 1080 to 64 × 64 pixels) can introduce some degree of noise or inaccuracies, we believe that this approach allowed us to maintain an adequate balance between the quality of the captured details and processing efficiency. This method was selected to ensure that critical patterns in the audio signals, which could be relevant for emotional classification, were retained despite the reduction in resolution.

It is important to note that this resizing process is not the only viable option. In fact, it is possible to generate the spectrograms directly at the desired final resolution (64 × 64 pixels) using tools such as librosa or matplotlib, which allow controlling the final size of the image while ensuring that relevant information is retained without the need for subsequent resizing. This alternative eliminates the intermediate step and can be equally effective, depending on the specific requirements of the analysis.

The choice of one approach over the other depends on the study’s objectives and the dataset’s characteristics. In our case, the decision to use resizing was motivated by the need to ensure that fine patterns and critical transitions, initially captured in high resolution, were adequately transferred to the lower resolution used for training the neural network.

Finally, by adding multiple sources of information to complement the images, an improvement in system accuracy was achieved. While spectrograms provide an overview of the energy and patterns in the voice signal, other features extracted directly from the audio can complement this information and enhance the model’s performance.

- **Normalization of spectrogram images**: The pixels of an image range from 0 to 255. These values are normalized to a range from 0 to 1 to facilitate their processing by the neural network. This normalization concludes the preprocessing. The resulting images serve as input for the network in the following stages, complemented by other selected features.

### 4.3. Feature Selection

Feature extraction is a fundamental pillar in voice emotion analysis. In this study, we lean towards widely recognized spectral features such as Mel-Frequency Cepstral Coefficients (MFCCs) and mel-spectrograms. Given the nature of our spectrogram images and the focus on deep learning techniques, we prioritize spectral features for several reasons. These features are effective at capturing relevant information in the audio signal that aligns with human sound perception. Additionally, the use of deep learning allows for further feature extraction throughout the process [64]. Spectral features and spectrogram images offer a detailed, comprehensive, and rich representation of audio signals. Spectral features are ideal for capturing complex frequency structures and providing noise robustness. In contrast, spectrogram images are intuitive representations that offer easier visualization and interpretability. These images can be processed using deep learning architectures such as CNNs, which have shown great success in image processing tasks and potentially lead to better performance in audio classification tasks.

Despite the popularity of prosodic and energy-based features in previous studies, we have chosen to focus exclusively on spectral ones for several reasons. Spectral features tend to capture more detailed frequency-related information, which is crucial for emotion recognition as different emotions can manifest in subtle changes in the frequency domain. While energy-based features like pitch and intensity are important, they can be more variable and less reliable across different speakers and recording conditions. Temporal features, on the other hand, might not capture the nuanced variations in the frequency domain that are critical for distinguishing between similar emotions. Moreover, spectral features align better with the deep learning techniques employed in our study, particularly convolutional neural networks (CNNs), which are adept at processing and extracting patterns from frequency–domain representations like spectrograms. Therefore, combining spectral features with spectrogram images provides a comprehensive approach that leverages both detailed frequency information and visual patterns, enhancing the overall performance of the emotion recognition system. Even though there are other techniques such as energy and waveform components that can be used in certain applications, they often lack the richness and robustness provided by spectral features and spectrograms for more complex audio analysis tasks.

We select five key features detailed below:

- **Zero-Crossing Rate (ZCR):** Reflects the rate of sign changes in the signal, representing how many times the signal changes between positive and negative values in a given interval. This feature is useful for identifying rapid changes in the audio signal, indicative of certain types of sound events, such as fricatives in speech, and can help differentiate between smooth and abrupt sounds associated with different emotional states.
- **Chroma STFT:** Represents the energy distribution for each chroma bin at specific time intervals. The chroma representation is useful for capturing harmony and tonal relationships in music and speech, which are relevant in emotion analysis because different emotions can affect the tonality and harmonic structure of speech.
- **MFCC:** These coefficients describe the power spectrum of a signal, being widely used in voice analysis since they align with human auditory perception. This is crucial for emotion analysis as emotions influence how we perceive voice.
- **Root Mean Square (RMS):** Denotes the average magnitude of the signal, calculating the square root of the mean value of the signal’s squares. RMS provides a measure of the signal’s energy, which can vary significantly with different emotions.
- **Mel-Spectogram:** Provides a logarithmic representation of frequencies, focusing on those most perceptible to the human ear. The mel-spectrogram is useful for visualizing how the signal’s energy is distributed across different frequencies over time and is particularly beneficial when combined with deep learning techniques, such as CNNs, to extract relevant patterns associated with different emotions.

#### Feature Scaling

**Figure 15:**
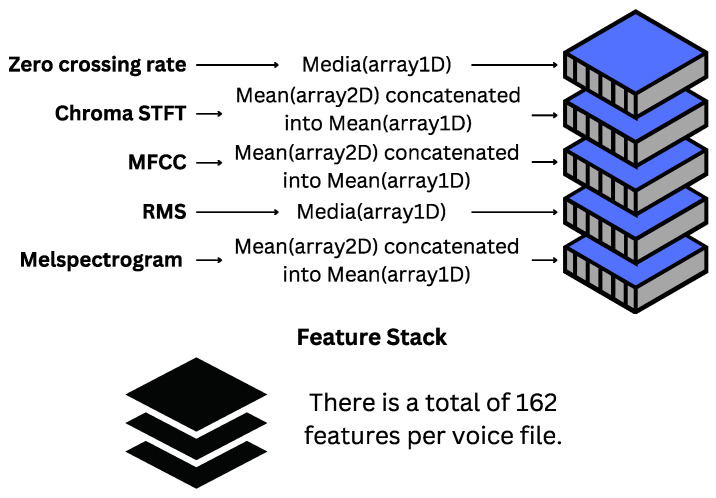
Process of obtaining spectral features.

After extracting the features, the next step involves normalizing them by eliminating their mean and scaling to unit variance. This process ensures that each feature contributes equally to the learning process and improves the model’s performance. Once normalized, these features are concatenated into a single one-dimensional matrix, resulting in a feature vector with 162 distinct values per audio file. This transformation is visually represented in Figure 15.

### 4.4. Feature Fusion

**Figure 16:**
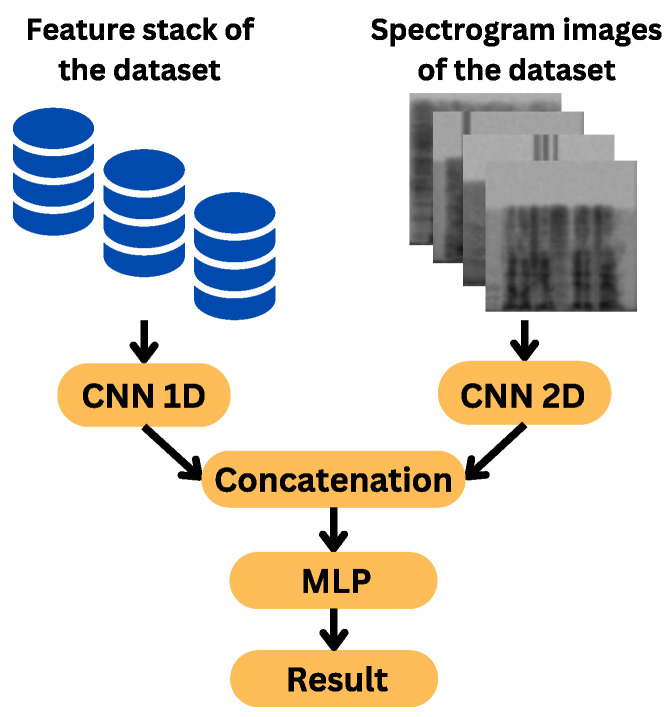
Feature fusion.

Two feature sets have been developed: spectral and image-based. From the five spectral features, a consolidated set of 162 features is obtained. In parallel, spectrograms are created from the voice files. The goal is to fuse both information sources using CNN and MLP. Although having multiple features does not guarantee better results, a specific neural network is used for each set. Both networks, one unidimensional for spectral features and another bidimensional for images, are trained separately. Each network extracts relevant features from its input data through several convolutional and pooling layers, ensuring the extraction of high-level abstractions from the raw data. These extracted features are then concatenated to form a unified feature vector, which effectively combines the diverse information from both sources. This vector is subsequently passed through a multilayer perceptron (MLP) for classification, enabling the model to learn complex patterns and relationships between the features. Figure 16 illustrates the comprehensive integration of these sources using a CNN and MLP. This approach ensures that the networks can effectively process and combine different types of data. Additionally, more sources such as video and images of the person in the interaction could be added, leading to a multimodal implementation and, in turn, a wide range of new applications and improved experience [65].

While attention mechanisms have become popular in recent research for their ability to dynamically focus on relevant parts of input data, we chose not to incorporate them in this study. Our decision was based on the observed effectiveness and efficiency of CNNs and MLPs in our empirical evaluations and the reviewed literature, as well as the desire to maintain computational simplicity and model complexity. Given the satisfactory results obtained and the well-established robustness of CNNs in handling SER tasks with personalized inputs (numerical values) [66], we focused on optimizing these techniques.

### 4.5. Classification

The introduction of deep learning to voice emotion recognition brought about significant improvements in error rate reduction [67]. Therefore, it is not surprising that for this final stage, three neural network models are designed and trained: a one-dimensional CNN, a two-dimensional CNN, and a combination of both. While there are pre-trained models and various network configurations, our goal is to propose a structure adapted to mixed inputs, spectral features and spectrogram images, prioritizing efficiency according to our data.

CNNs are chosen for their ability to handle variability in voice signals, such as differences in tone and pronunciation. This advantage extends to both spectrogram images and spectral features. While many studies opt for MLP networks, evidenced by studies like the one proposed by Lee and Kim [45], which achieve good results with spectral features, our choice of a one-dimensional CNN seeks to allow the model to discover abstract patterns during its training, which could positively influence its performance. However, emotion recognition remains a dynamic process and a complex task, as each person expresses emotions differently [68].

#### Neural Network Configuration

Neural network design involves hyperparameter tuning, which can be approached mathematically or empirically. Although experimentation can be practical, it is not always ideal for model validation. Using pre-trained networks like ResNet or VGG-19 can be helpful, but they are designed for images with recognizable objects, not spectrograms. Therefore, designing a network from scratch is valid, especially when handling multiple information sources.

Regarding the empirical methods, it is important to note that the recommendations arising from these practices come from previous experience. For example, it has been observed that 1–2 hidden layers may be suitable for low-dimensional data, while 3–5 layers are suggested for high-dimensional data. In addition, the use of powers of 2 for the number of nodes, such as 16, 32, 64, is common due to the computational efficiency they provide. A larger number of nodes implies higher model complexity and longer training duration but allows the model to learn deeper features. On the other hand, fewer nodes speed up training time, reduce complexity, and decrease the ability to learn deep features. While these empirical recommendations may be useful, they do not guarantee optimal hyperparameter tuning.

For more meticulous hyperparameter selection, specialized tools are employed to optimize the network configuration by exploring variations in the hyperparameters. This includes tuning the number of hidden layers, the units in dense layers, the filters in convolutional layers, the activation function, the optimizer, the learning rate, and the number of epochs. This tuning phase is applied to the CNN-1D and CNN-2D networks and their concatenation to integrate with an MLP. Despite this optimization, the consideration of using node sizes in powers of 2 is maintained for its computational benefits on the GPU, in line with a trend widely observed in previous research.

**Figure 17:**
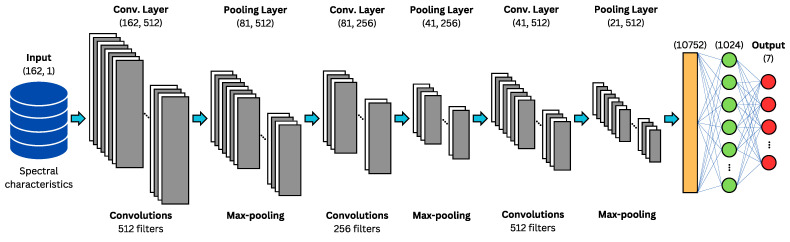
CNN-1D architecture for spectral features.

**Figure 18:**
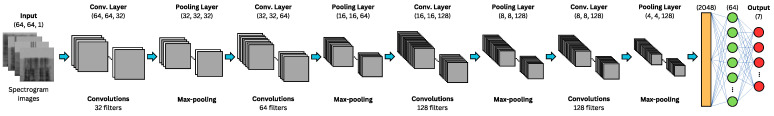
CNN-2D architecture for spectrogram images.

**Figure 19:**
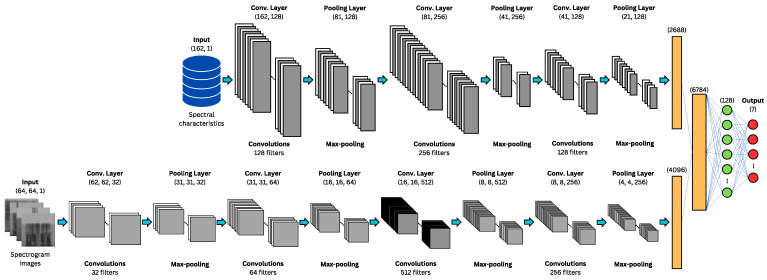
Combined CNN-1D and CNN-2D architecture with MLP classification.

The development for each part of the concatenated architecture is shown below:

- **Configuration of CNN-1D for Spectral Features:** The CNN-1D is used for handling spectral features, with a structure comprising three convolutional layers, each followed by a MaxPooling1D layer and a dropout regularization layer to prevent overfitting. This model is specifically designed to process the 162 spectral features. A multilayer perceptron (MLP) is used as the final classifier for this network. Hyperparameter tuning was performed using specialized tools and manually. A graphical representation of this architecture is provided in Figure 17, where the input consists of spectral features, gray represents the convolutional and pooling layers, orange the flattening layer, green the dense layers, and red the output layer.
- **Configuration of CNN-2D for Spectrogram Images:** The CNN-2D is designed to process 64 × 64 grayscale spectrogram images. This structure comprises four convolutional layers, each followed by a MaxPooling2D layer and dropout regularization layers to minimize overfitting. This model handles the spectrogram images independently and also uses an MLP as the final classifier. Hyperparameter tuning was performed using specialized tools and manually. A graphical representation of this architecture is provided in Figure 18, where the input consists of spectrogram images, with gray representing the convolutional and pooling layers, orange for the flattening layer, green for the dense layers, and red for the output layer.
- **Configuration of Combined CNN-1D and CNN-2D:** The combined model integrates both CNN-1D for spectral features and CNN-2D for spectrogram images. The features extracted by these networks are concatenated and then passed through a multilayer perceptron (MLP). This final model leverages the strengths of both individual models to improve overall performance. Hyperparameter tuning was performed using specialized tools. A graphical representation of this concatenated architecture is provided in Figure 19, where the input consists of spectral features and spectrogram images, gray represents the convolutional and pooling layers, orange highlights the two flattening layers (one for each network) and the concatenation layer, green denotes the dense layers, and red corresponds to the output layer.

### 4.6. System Design

**Figure 20:**
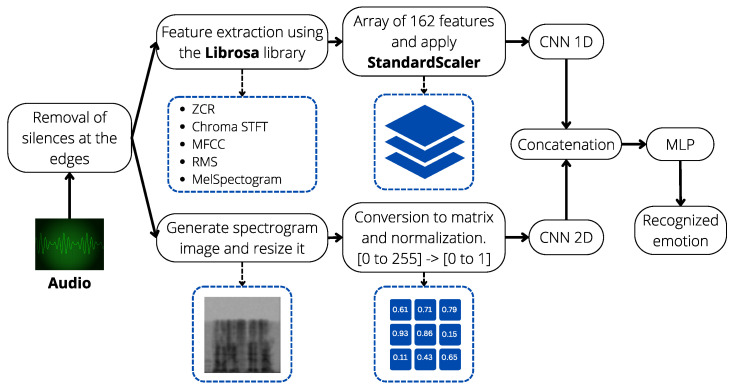
Block diagram of the system design.

The system integrates all the components described in this section. A visual summary of the design is shown in Figure 20. For more details on the integrated system, please refer to the preprocessing, feature selection, and classification stages. In this link, https://github.com/Roly2340l/EmoDSc-SER (accessed on 3 November 2023), the process of each stage prior to the training of the model and also the configuration of the neural networks are detailed.

## 5. Results

In the search for accurate models for speech emotion recognition, this study experimented with various deep learning architectures, evaluating their capacity and effectiveness using different datasets and feature sources.

Initially, an evaluation of a model using a CNN-1D coupled with an MLP is undertaken. This architecture is oriented towards processing spectral voice features, leveraging the richness of information these parameters offer in detecting emotional nuances. Subsequently, aiming to explore the visual potential of emotions in the spectrogram, a model based on the architecture of a CNN-2D coupled with an MLP is designed. This approach allows analyzing spectrogram images, considering the depth and texture of emotions reflected in the frequency and time domain.

For the training of these models, the EmoDSc database elaborated in the context of this work (presented in Section 4.1) is used. Recognizing the uniqueness and strength of both architectures, they are merged into a hybrid model that concatenates both CNN-1D and CNN-2D, culminating in an MLP. This combination aims to bring together the strengths of both feature sources, enhancing emotion recognition capabilities. Finally, to test the versatility and adaptability of the combined architecture, it was trained with two well-known databases, EMOVO [69] and MESD [70]. These databases, with variations in quantity and labels, served as a robustness test for the proposed architecture, demonstrating its ability to adjust to different datasets.

To evaluate the effectiveness of a model, several metrics are considered, such as accuracy, recall, and F1-score. Since there are multiple possible outputs, we use the weighted average (WA) for each metric. In this case, WA is equivalent to a simple average by having the database balanced.

In the following subsections, we detail the results derived from each evaluation. We evaluate the effectiveness, accuracy, and potential of these architectures, and also analyze the overall performance of the proposed system throughout the various research phases.

### 5.1. Model 1—Spectral Features Only

**Figure 21:**
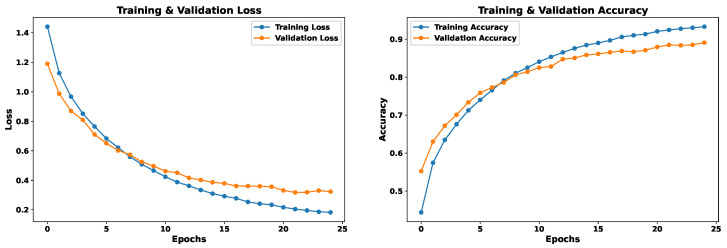
Loss and accuracy graphs for the training and validation set (Model 1).

This model uses as input 162 features extracted from the voice signal, as detailed in Section 4.3. The EmoDSc dataset was divided into three parts, 80% for training, 10% for validation, and 10% for testing, ensuring a representative class distribution. Consequently, a one-dimensional CNN is used. Figure 21 presents the loss and accuracy graphs throughout the training process with our EmoDSc dataset. This model achieves an accuracy of 93.29% in training (with the 80% of EmoDSc) and 89.10% in validation (with the 10% of EmoDSc data not seen in the training process). From the sixth epoch onwards, training and validation accuracies tend to converge, as do their respective losses. This suggests that, as the number of epochs increases, the model begins to show signs of overfitting. Although the training accuracy reaches 92.42%, and the gap with the validation set is not significant, a higher number of epochs could exacerbate this overfitting.

**Figure 22:**
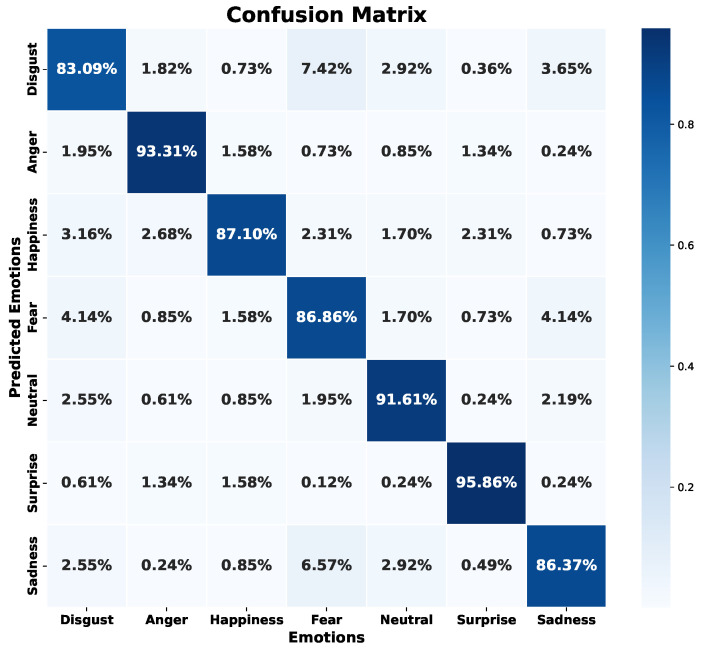
Confusion matrix for the test set (Model 1).

Figure 22 shows the confusion matrix obtained during the validation phase. The model has difficulties identifying the emotion “disgust”, and to a lesser extent, the emotions of “sadness”, “fear”, and “happiness”. In contrast, emotions like “anger”, “neutral”, and “surprise” show superior results, with success rates above 83%, reaching more than 91% in these emotions.

Regarding the error metrics shown in Table 4 and Table 5, the emotion “fear” exhibits the lowest accuracy in validation with 79% and an accuracy of 82% in the test set. Similarly, “disgust” shows 85% accuracy in both validation and test sets. However, the recall for “disgust” is 80% in validation and 83% in testing, while for “fear” it is 87% in both, surpassing its accuracy. In contrast, the emotions “anger”, “neutral”, and “surprise” maintain a balance between accuracy and recall, both above 90% across validation and test sets. Although the emotion “happiness” has an accuracy of 92% and 87% recall in validation, it maintains the same recall in the test set, indicating consistency. This suggests that, although there are many hits in terms of accuracy, the recall indicates a significant number of false negatives, confusing audio files that correspond to “happiness” with another emotion. Therefore, recognition difficulties are identified in four particular emotions: “disgust”, “sadness”, “happiness”, and “fear”.

The convergence of the training and validation curves indicates that the model is not significantly underfitting. However, the slight widening gap between the training and validation accuracies towards the end of the training process suggests mild overfitting. The model exhibits low bias as it achieves high accuracy on the training set, indicating it captures the complexities in the data. Regularization techniques and careful parameter tuning could be explored to further reduce overfitting and enhance model efficiency.

Although this model faces challenges in identifying certain emotions, particularly “fear” and “disgust”, it demonstrates good performance in identifying the rest of the emotions. The WA accuracy achieved by this model (i.e., 89%) surpasses results from research presented by Lee et al. [9] and Lee and Kim [45]. Notably, the highest accuracy recorded in the study by Sun [16] was 85.12% for the EMODB database, even though these studies used advanced deep learning techniques, such as DNN, CNN, or R-CNN. However, despite these outstanding results, overfitting could pose a problem, especially when introducing new data to the model. Therefore, it cannot yet be considered a fully efficient model.

### 5.2. Model 2—Spectrogram Images Only

**Figure 23:**
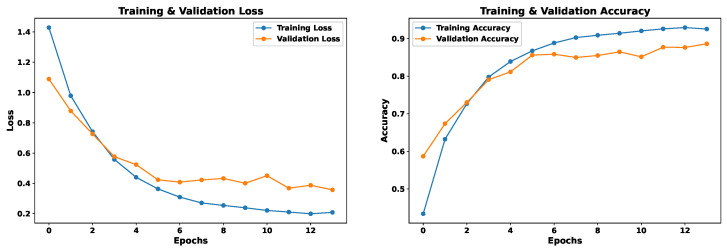
Loss and accuracy graphs for the training and validation set (Model 2).

For this model, spectrogram images in the form of matrices are used as input. The EmoDSc dataset was divided into three parts, 80% for training, 10% for validation, and 10% for testing, ensuring a representative class distribution. Figure 23 presents the graphs of loss and accuracy for the training and validation sets. After three epochs, both training and validation accuracy converge to an optimal point. With a higher number of epochs, accuracy reaches 88.62% in validation and 92.55% in training. This model shares a similar difficulty to the one based on spectral features: evident overfitting. Although it is not very pronounced, with more epochs, the model would begin to memorize specific patterns from the dataset.

**Figure 24:**
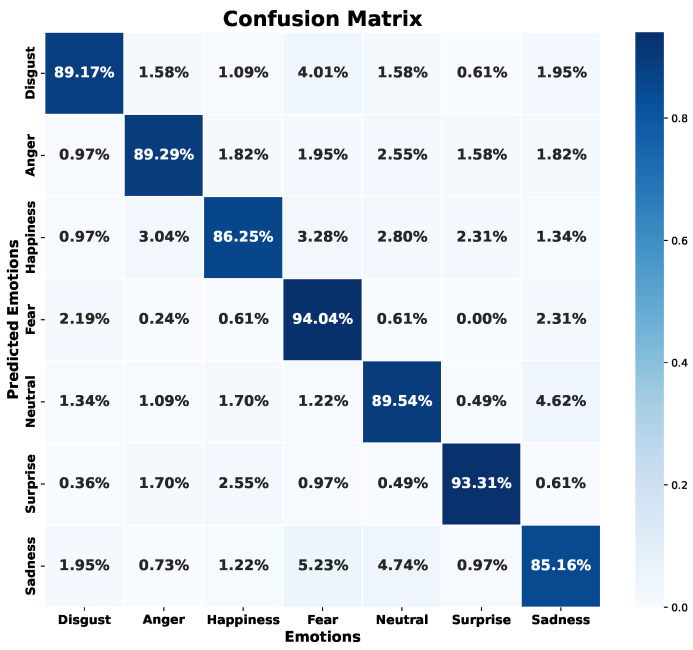
Confusion matrix for the test set (Model 2).

When examining predictions with a test set, as seen in Figure 24, the emotions “sadness” and “happiness” have the lowest success rates. However, in contrast to the first model, the recall for the rest of the emotions exceeds 89%, with emotions like “fear” and “surprise” even surpassing 93% accuracy.

The error metrics in Table 6 and Table 7 reveal that, in terms of recall, the best result is obtained with the “fear” emotion, with a recall of 92% in validation and 94% in testing. However, in accuracy, it is the emotion “surprise” that stands out, with 94% accuracy in both validation and test sets. The emotion “fear”, despite having only 85% accuracy, has the highest recall, indicating that the model adequately identifies the emotion but tends to confuse other emotions with it. This contrasts with the model trained with spectral features. On the other hand, this model shows a less accurate prediction of the emotion “neutral” (88% accuracy in validation and 90% in testing) compared to other emotions. Like the first model, this one also has a somewhat low recall for the emotion “happiness” (83% in validation and 86% in testing), despite having high accuracy (91% in both validation and testing).

The convergence of the training and validation curves indicates that the model does not exhibit significant underfitting. However, the small gap between the training and validation accuracy curves in later epochs suggests some degree of overfitting. This overfitting is not very pronounced, but it is more evident than in the first model (spectral features only), which could lead the model to start memorizing specific patterns from the dataset with more epochs. The model exhibits low bias as it achieves high accuracy on the training set, indicating it captures the complexities of the data. The small gap between the training and validation accuracies indicates moderate variance, suggesting that the model generalizes well to unseen data.

Although this model yields similar results to the previous one, it shows overlapping trends in the emotions of “fear” and “happiness” and some notable differences, such as those observed in “disgust” and “neutral”. Moreover, in general, this model has a slightly higher accuracy compared to the previous model. Thus, each model, depending on the feature source, has its “starring” emotions. Nevertheless, the first and the second model present a similar WA in the evaluated metrics.

Comparing this model with other studies, such as those developed by Lee and Kim [45] and Zhang et al. [48], the WA accuracy achieved (i.e., 90%) surpasses their results, and it is slightly below the 90.78% achieved by Meng et al. [62]. In contrast, Liu et al. [41] and Mustaqeem and Kwon [43] achieved a maximum accuracy on emotions such as “happiness” of 98.51%. However, these studies use different databases, and most of them are based on voice signal features, so they are only reference points.

### 5.3. Model 3—Concatenated Features

As noted above, each model based on its own trait source showed particular results, suggesting complementarity between them. Some emotions that showed low success rates in one model excelled in the other. Under this premise, we propose to merge both feature sources. We concatenate both neural networks that worked independently with their feature source. The final result aims to cover the weaknesses that each model had independently, so that it obtains results with a balanced accuracy for each emotion.

**Figure 25:**
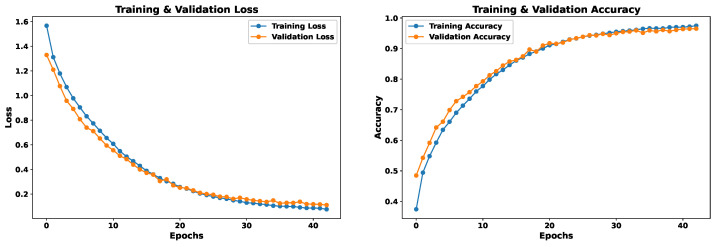
Loss and accuracy graphs for the training and validation set (Model 3).

The EmoDSc dataset was divided into three parts, 80% for training, 10% for validation, and 10% for testing, ensuring a representative class distribution. Figure 25 presents the accuracy and loss graphs for the training and validation set. For training, the accuracy reached is 97.48%, while for validation it is 96.51%. Unlike previous models, the combination of images and spectral features achieves a WA accuracy of 97%, superior to any other presented model and previous works. Furthermore, overfitting is practically non-existent. In fact, by reducing the number of epochs, accuracy would still be around 96%. Similar studies, like the ones presented by Liu et al. [41], which fused various feature sources, reported excellent accuracy results. However, our model achieved marginally better outcomes.

**Figure 26:**
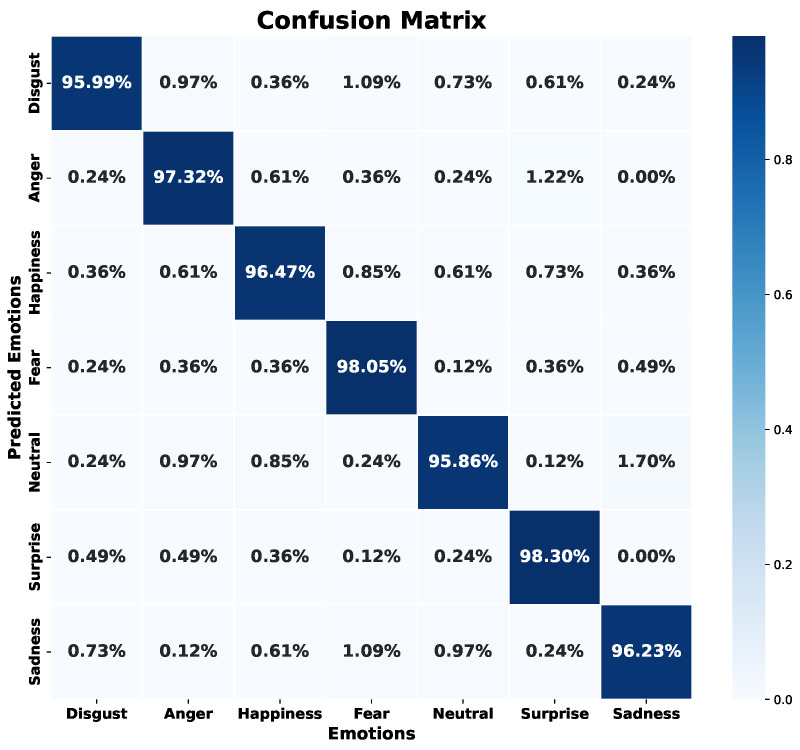
Confusion matrix for the test set (Model 3).

Figure 26 shows the confusion matrix with the test set, reflecting superior performance compared to previous models and indicating that the model is robust against previously unknown data as long as they are of the same nature. Additionally, the model achieves success rates above 95.86% for each emotion. The emotion “neutral” also experiences a significant improvement. In general, the emotions “neutral”, “disgust”, and “sadness” are the ones that show slightly lower success rates, but in this model, the results are more homogeneous, achieving a remarkable balance.

Table 8 and Table 9 reflect these positive results. Accuracy exceeds 94% for all emotions in both the validation and test sets, with a WA of 96% in validation and 97% in testing. Additionally, recall exceeds 94% in all cases. The homogeneity of the results by emotion suggests an ideal balance, whether due to coincidence or the success of feature fusion. The initial idea was that fusion would improve overall accuracy, and this assumption has been confirmed with the results.

The fusion of spectral features and spectrogram images has resulted in a remarkable improvement in the model’s performance. Each feature source, when used separately, showed significant limitations, with varying accuracy and recall depending on the emotion. However, combining both has overcome these limitations, achieving a more balanced and robust model.

Analyzing the training and validation curves reveals that the model does not suffer from underfitting, as the curves converge adequately. Nonetheless, there is a slight indication of overfitting in the later epochs, suggesting that regularization techniques could be beneficial to further enhance the model’s generalization capabilities. Despite this minor overfitting, the difference between training and validation accuracies is minimal, indicating moderate variance and good generalization to unseen data.

The performance metrics obtained are impressive. With accuracy and recall exceeding 94% in most cases for the validation and test sets, the model proves to be both precise and sensitive. High F1 scores indicate a balance between accuracy and recall, which is crucial for practical applications where errors can be critical.

In summary, the combination of spectral features and spectrogram images has allowed the development of a robust and accurate model, capable of overcoming the limitations of each feature source separately and offering superior performance in emotion classification.

### 5.4. Neural Network Architecture with Other Databases

Model 3 fuses spectral features and spectrogram images achieving high accuracy with our built EmoDSc (see Section 4.1), which ensures the same nature of the data. Although it has shown good results with the general base, to validate its performance, we carried out more experiments with two well-known databases: EMOVO [69] and MESD [70]. The network architecture is evaluated with data in Italian and Spanish. Each database is trained individually, considering that they present different emotions. Both datasets were recorded in controlled environments, meaning the emotions were acted out. Detailed information about the EMOVO and MESD databases is provided in Table 10.

#### 5.4.1. EMOVO Dataset

This database consists of 588 audio files and seven emotions, equivalent to 84 audio files per emotion. The corresponding preprocessing was applied to adapt the audio files to the network inputs. With the data augmentation technique, 2352 audio files are obtained, distributing 80% for training, 10% for validation, and 10% for tests.

**Figure 27:**
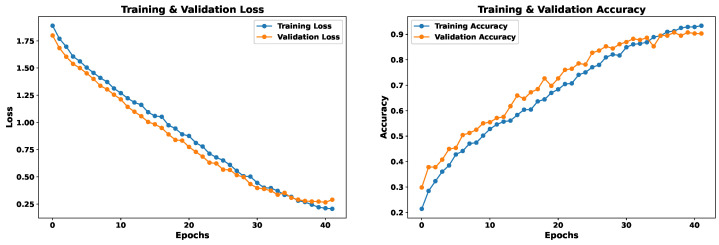
Loss and accuracy graphs for the training and validation set (EMOVO).

Figure 27 shows the loss and accuracy graphs, reaching an accuracy of 90.34%. However, training accuracy reaches 93.39%, indicating overfitting. The ideal epoch to stop training would be the 36th, when both accuracies are around 89.5%.

**Figure 28:**
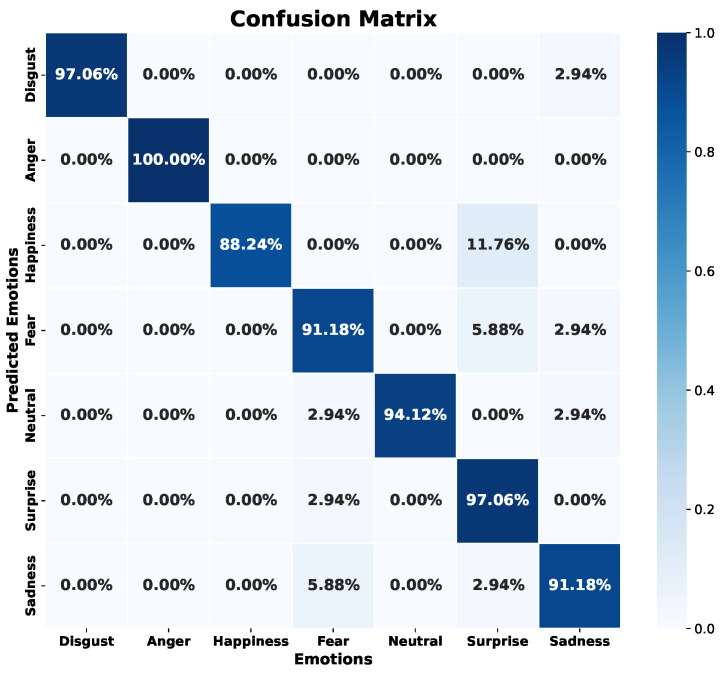
Confusion matrix for the test set (EMOVO).

The test set, although small, is of the same nature as the training set. Figure 28 shows the confusion matrix with good results, especially for emotions “disgust”, “surprise”, and “anger”. Although these results might suggest some overfitting, adjusting the number of epochs could alleviate this problem.

As observed in Table 11, the error metrics for the validation set are quite high in terms of accuracy and recall, although the emotion “surprise” presents some difficulties. Emotions such as “anger” and “sadness” achieve very high accuracy and recall, suggesting that the model handles these categories well. The weighted average (WA) accuracy for the validation set is 91%, indicating good overall performance.

In Table 12, the error metrics for the test set also show high results in terms of accuracy and recall, although again “surprise” presents difficulties. Emotions such as “anger”, “disgust”, “happiness”, and “neutral” achieve 100% accuracy, which could indicate overfitting due to the smaller dataset size compared to the EmoDSc dataset. The weighted average (WA) accuracy for the test set is 95%, which is very high but may indicate a model that has learned the specific characteristics of this smaller dataset too well.

Overfitting is evident in the discrepancy between the training and validation accuracy curves towards the later epochs. This suggests that the model is memorizing specific patterns from the training set, reflected in the high accuracy in the test set, especially for “anger”, “disgust”, “happiness”, and “neutral” with 100% accuracy. The convergence of the loss and accuracy curves indicates that the model does not suffer from underfitting.

The reduced size of the Italian dataset (EMOVO), compared to the original English database, limits the model’s ability to learn from a diverse set of examples, increasing the likelihood of overfitting. Although the model shows low bias and high accuracy on the training set, a smaller dataset can lead to excessive fitting to the peculiarities of those data, reducing its generalization capability.

The small difference between the training and validation accuracies suggests moderate variance, indicating that the model generalizes reasonably well to unseen data. However, the discrepancy in the “surprise” emotion indicates that some emotions are harder to generalize than others. In conclusion, with the EMOVO database, encouraging results were obtained, similar to the main database of the study. However, due to its limited size, overfitting is a problem. Although adjusting the architecture could mitigate this effect, the main purpose is to validate the performance of the current architecture. It is advisable to use a large, varied, and information-rich database.

#### 5.4.2. MESD Dataset

**Figure 29:**
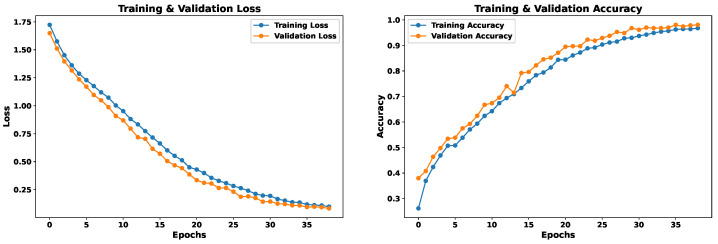
Loss and accuracy graphs for the training and validation set (MESD).

This database has 1150 audio files distributed in six emotions, which translates to a range of 191 to 192 audio files for each emotion, distributing 80% for training, 10% for validation, and 10% for tests. The relevant preprocessing is carried out so that the data fit the neural network inputs. This preprocessing process results in a total of 4600 audio files. In Figure 29, the loss and accuracy graphs show that, unlike the EMOVO database, the MESD achieves a WA accuracy of 96% with the neural network that fuses features. Notably, there does not seem to be overfitting. This lesser overfitting, or its absence, could be explained due to the larger amount of information that the database presents, added to the fact that it contains fewer emotions but more audio files per emotion. The accuracy during training was 96.76%, while for validation it reached 98.07%.

**Figure 30:**
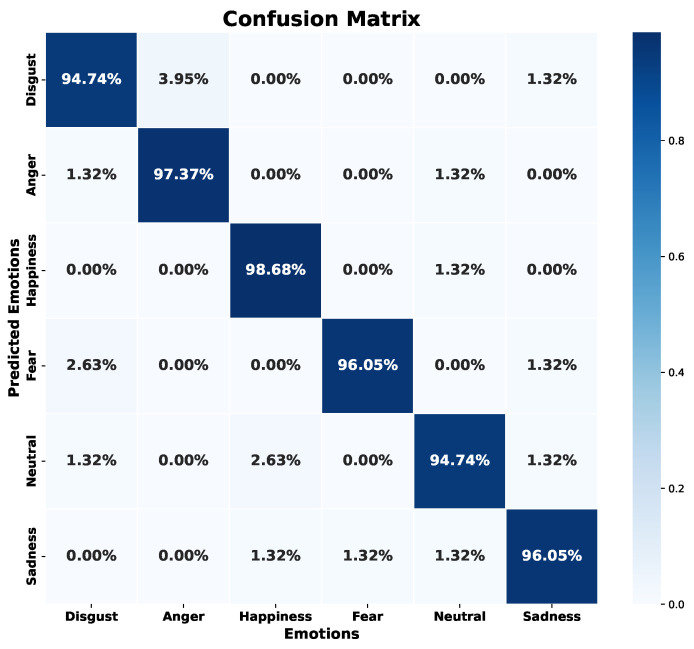
Confusion matrix for the test set (MESD).

Regarding the test set, the success rates are high, exceeding 94.74% for each emotion, as reflected in Figure 30. Unlike the results obtained with the EMOVO database, these are more homogeneous in terms of success rate per emotion and do not show signs of overfitting, thus providing more reliable results.

Table 13 presents the error metrics for the validation set, showing results above 95% in terms of accuracy and recall for each emotional state, with a weighted average of 98%. In particular, emotions such as “happiness”, “neutral”, “surprise”, and “sadness” exhibit accuracy and recall of 99% or higher, indicating high model performance in the validation phase.

Similarly, Table 14 presents the error metrics for the test set, also showing results above 95% in accuracy and recall for each emotion, achieving a weighted average of 96%. Emotions like “fear” reach accuracy levels close to 99%, suggesting that the model generalizes well to unseen data.

With the MESD database, more promising results are achieved than with EMOVO. Although this is partly due to the larger amount of data and the lower variety of emotions, it is essential to highlight that the neural architecture designed for feature fusion proves to be efficient, regardless of the database with which it works. However, the model shows signs of overfitting, evidenced by the discrepancy between the training and validation accuracy curves in the later epochs.

Despite this, the convergence of the loss and accuracy curves indicates that the model does not suffer from underfitting and can learn the underlying patterns in the training data. However, the small dataset size may lead to low bias but moderate variance, as the model generalizes reasonably well, though not perfectly, to unseen data.

In conclusion, the model exhibits slight overfitting. The reduction in the number of emotions and the limited amount of data affect the model’s ability to fully generalize to real-world scenarios. However, the architecture is still efficient, which makes it a valuable architecture for other related projects, positioning it as a starting point. However, it is advisable to opt for voluminous databases, as this helps to avoid overfitting and facilitates the extraction of the maximum number of features from spectral sources and spectrogram images.

## 6. Discussion

Understanding emotions and their recognition across various modalities is essential for the correct implementation of a speech emotion recognition system. The difference between “voice” and “speech” and the importance of feature pre-selection highlight the complexity present in speech emotion recognition.

Throughout this study, we have emphasized the significance of rigorous preprocessing and appropriate feature selection, as there is no single standard set used universally. Spectral features and spectrogram images, while powerful individually, show limitations when evaluated separately, reflecting the complex and multidimensional nature of emotion recognition. Specifically, spectral features struggle with identifying “disgust”, but excel with “surprise”. Conversely, spectrogram images are accurate for “fear”, “surprise”, and “disgust” but less so for “happiness”. These divergences illustrate that not all features are equally informative for all emotions.

Our research highlights the milestone achieved through feature fusion. By integrating spectral features and spectrogram images, we combine their strengths, leading to substantial improvements in accuracy and recall, with our fused model achieving an accuracy exceeding 96%. This synergy proves decisive, outperforming individual models significantly.

The proposed neural network architecture, comprising a CNN-1D, CNN-2D, and MLP, demonstrates consistent performance regardless of the training database, providing a solid foundation for future research. Each component, from data preparation and feature selection to the classification architecture, contributes crucially to the comprehensive emotion recognition system.

A critical aspect emerging from our results is the importance of the volume and quality of the database. The EmoDSc dataset, with its extensive and balanced distribution of audio files, serves as the backbone for our experiments, enabling superior performance and reduced overfitting. This underscores the need for extensive and diversified databases in future emotion recognition projects.

The results have significant practical implications. Models with this level of accuracy have vast potential in fields such as mental health, human–machine interaction, and advertising. However, real-world data may differ from training data, and the model’s effectiveness will depend on its generalizability, the application context, and the quality of recording hardware.

Another challenge is the recognition of emotions across different languages and dialects. Our current model has been primarily trained and tested on English datasets, with additional validation on Italian and Spanish datasets. However, emotions can be expressed differently across cultures and languages, which may affect the model’s accuracy. Future research should explore the adaptability of the model to various linguistic contexts and investigate techniques to improve its performance in multilingual and multicultural environments.

Furthermore, the quality of the recording hardware used in real-world applications can significantly impact the performance of the emotion recognition system. Variations in microphone quality, background noise, and recording conditions can introduce noise and affect the accuracy of the model. Future studies should consider robust preprocessing techniques and noise reduction methods to mitigate these issues.

Lastly, while our proposed model shows high accuracy, it is essential to conduct extensive real-world testing to validate its practical applicability. Implementations in different domains, such as healthcare, human–robot interaction, and customer service, will provide valuable insights into the model’s effectiveness and areas for improvement.

In summary, our findings demonstrate that combining multiple feature sources and using comprehensive databases can significantly enhance the accuracy and robustness of speech emotion recognition systems, paving the way for future advancements in this field.

## 7. Conclusions

In this work, a neural network architecture for speech emotion recognition is proposed. Our architecture integrates a CNN-1D, a CNN-2D, and an MLP, using two feature sources: spectral features and spectrogram images. This configuration aims to increase efficiency in terms of accuracy, mitigating the weaknesses that would arise from using the sources independently. Feature fusion proved to be decisive, leading the fused model to significantly outperform the individual models, with accuracy exceeding 96%. The relevance of the volume and quality of the database used also emerge from the results, suggesting the importance of extensive and diversified databases in future emotion recognition projects. In this sense, a consolidated dataset, called EmoDSc, is formed by amalgamating several databases, resulting in a diversified and information-rich database.

While the combination of spectral features and spectrogram images has proven to be robust, we consider that other elements could be incorporated to further improve performance. This suggests exploring more advanced machine learning techniques, studies of correlation between extracted features, and the potential to include additional data, such as facial and body gestures, in search of multimodal emotion recognition.

The consideration of emotion intensity, pure or overlapping emotions, and emotions captured in real life scenarios is indeed crucial. These aspects represent significant directions for future research, as they reflect the complexities inherent in human emotions. Addressing these complexities, particularly through the application of our techniques to “in-the-wild” datasets, will allow us to tackle the challenges posed by more naturalistic settings. Future research will also delve into aspects related to database integration, such as incorporating more nuanced emotion annotations, including overlapping emotions, and evaluating annotator agreement.

Looking ahead, we plan to implement this architecture in social robot systems, equipping robots with advanced emotion recognition capabilities, thereby enhancing human–robot interaction and broadening the applicability of our research in real-world settings.

## Figures and Tables

**Table 2:** Stages used for the development of a recognition model.

Work (Year)	Pre-Processing	Source of Characteristics	Classification
Harár et al. [15] (2017)	Audio Silence Removal and Normalization	Spectral and Prosodic Traditionals	DNN
Liu et al. [41] (2018)	Raw audio signal used	Spectrogram images and hyper-prosodic features	CNN, DNN
Zhao et al. [59] (2018)	Raw audio signal is used, Normalization	Crafted or constructed features, which go through a Deep Feature Learner, with LFLB and LSTM	CNN LSTM Network
Lee et al. [9] (2019)	Elimination of silences at the extremes	Spectral and prosodic traditionals	SVM, DNN
Han and Wang [21] (2019)	Sampling, quantization, pre-emphasis, framing, window addition and endpoint detection	Traditional prosodic and extracted via DBN	PSVM
Meng et al. [62] (2019)	Raw audio signal is used	Traditional spectral and mel-spectrogram imaging	Multi-CNN dilated
Mustaqeem and Kwon [43] (2019)	Removing noise and quiet parts in audio	Spectrogram images	CNN, DSCNN
Yoon et al. [55] (2019)	Raw audio signal is used	Traditional spectral and prosodic. It is multimodal, so it includes text	RNN
Li et al. [50] (2019)	Standardization of duration to 7.5 s, 800-length and 400-skip windowed	Spectrogram images	CNN-BLSTM
Wu et al. [58] (2019)	2 s spectrogram splitting	Spectrogram images	Capsule Networks
Xie et al. [53] (2019)	Raw audio signal is used	Frame-level features based on ComParE openSMILE	LSTM
Lee and Kim [45] (2020)	Normalization	Traditional spectral, mel-spectrogram imaging	MLP, CNN
Sun [16] (2020)	Raw audio signal used	Raw audio signal and genre information used	R-CNN
Zhang et al. [48] (2020)	Scaling and segmentation	Mel-spectrogram images are used, in three different forms, such as 1D, 2D and 3D	Multi-CNN
Aouani and Ayed [42] (2020)	Raw audio signal is used	Traditional spectral, temporal and prosodic. An Auto Encoder is applied for final parameter selection	SVM
Issa et al. [54] (2020)	Raw audio signal is used	Spectral traditional	CNN
Mustaqeem et al. [49] (2020)	Division of the audio into segments, grouping by K-means Clustering, sequencing of the selected segments as keys, application of STFT and obtaining spectrograms	Extracted using CNN ResNet	Bidirectional LSTM
Pappagari et al. [11] (2021)	Normalization	Spectral Traditionals	ResNet
Cai et al. [2] (2021)	It uses the raw audio signal	Traditional spectral, and by means of 1D CNN, 2D CNN and RNN. It is for multimodal use	DNN
Wang and Han [52] (2021)	Sampling, quantization, pre-emphasis, framing, window addition and end-point detection	Extracted by DBN	Shallow Neural Network
Graterol et al. [56] (2021)	Conversion of raw data to representations suitable for emotion analysis	In audio, normalized spectrograms. Although it also uses text and feature vectors extracted from video and image	Transformer architecture and meta-learning with MLP
Abdelhamid et al. [10] (2022)	Data augmentation by noise addition	Log-Mel spectrogram imaging	CNN LSTM Deep Network
Heredia et al. [57] (2022)	Raw audio signal used; however, image information is obtained	An MFCC feature extractor is used for audios. Additionally, they obtain text from audios and faces from images	EmbraceNet+
Singh et al. [46] (2023)	Data augmentation by adding noise, Normalization	Multiple spectral and rhythmic features were tested, including ZCR, Chroma-Stft, MFCC, RMS, Mel, Tempogram, Fourier-tempogram, among others	Combination of CNN-2D and LSTM with an attention layer
Mishra et al. [47] (2023)	Normalization	Different combinations of $MFCC_{mean}$, $MFCC_{S}E$ and $MFCC_{A}E$	DNN
Sha et al. [51] (2024)	Raw audio signal used	Use of the time-frequency convolution module (TFCM) to extract interaction information in the time-frequency domain, MFCC and Hybrid Dilated Convolution (HDC)	Residual Attention Multi-Layer Perceptron Proposal (RA-GMLP)
Lin et al. [60] (2024)	Raw audio signal used	Uses acoustic features from mel-spectrograms, extracted with a 32 ms window and 16 ms hop (50% overlap). Applies Z-normalization with parameters estimated from the training set.	CNN-regressor, Temp-GRU, Temp-CNN, Temp-Trans and Temp-Triplet
Deshmukh et al. [44] (2024)	Silence removal, Noise Removal and Normalization	Pitch and frequency features are calculated such as Chroma-Stft, Roll-off, Spec_cent, Spec_bw and ZCR are calculated from speech.	Probabilistic Neural Network (PNN), Recurrent Neural Network

**Table 3:** Error metrics used and recognition results.

Work	Databases	Results
Harár et al. [15] (2017)	EMODB (Subset, only Anger, Neutral and Sadness)	Accuracy: 96.97%
Liu et al. [41] (2018)	CASIA	Accuracy: Max (Happiness): 98.51%, Min (Neutral): 93.91%
Zhao et al. [59] (2018)	EMODB, IEMOCAP	Accuracy: EMODB: 95.33%, IEMOCAP: 89.16%
Lee et al. [9] (2019)	RAVDESS	Accuracy: A: DNN: 68.50%, SVM: 35.60%
Han and Wang [21] (2019)	Proprietary	Error rate: 5.00%
Meng et al. [62] (2019)	IEMOCAP, EMODB	Accuracy: IEMOCAP: 74.96%, EMODB: 90.78%, Combined: 63.84%
Mustaqeem and Kwon [43] (2019)	IEMOCAP, RAVDESS	Accuracy: IEMOCAP with CNN: 84.00%, RAVDESS with CNN: 80.00%. IEMOCAP with DSCNN: 84.00%, RAVDESS with DSCNN: 81.00%
Yoon et al. [55] (2019)	IEMOCAP	Accuracy: 63.00%
Lee et al. [50] (2019)	IEMOCAP	Accuracy: WA: 81.60%, IA: 82.80%
Wu et al. [58] (2019)	IEMOCAP	Accuracy: 72.73%
Xie et al. [53] (2019)	CASIA, eNTERFACE, GEMEP	Accuracy: CASIA: 92.80%, eNTERFACE: 89.60%, GEMEP: 57.00%
Lee and Kim [45] (2020)	Emotion01	Accuracy: MLP: 75.00%, CNN: 60.00%, CNN: 60.00%
Sun [16] (2020)	CASIA, EMODB, IEMOCAP	Accuracy: CASIA: 84.60%, EMODB: 90.30%, IEMOCAP: 71.50%, IEMOCAP: 71.50%
Zhang [48] (2020)	AFEW5.0, BAUM-1	Accuracy: AFEW5.0: 35.77%, BAUM-1: 44.06%
Aouani and Ayed [42] (2020)	RML emotion database (English language only)	Accuracy: 74.07%
Issa et al. [54] (2020)	RAVDESS, IEMOCAP, EMODB	Accuracy: RAVDESS: 71.61%, EMODB: 95.71%, IEMOCAP: 64.30%
Mustaqeem et al. [49] (2020)	IEMOCAP, EMODB, RAVDESS	Accuracy: IEMOCAP: 81.01%, EMODB: 91.14%, RAVDESS: 82.01%
Pappagar [11] (2021)	MSP-Podcast, Crema-D, IEMOCAP	F1-score: MSP-Podcast: 56.44%, Crema-D: 76.60%, IEMOCAP: 56.69%
Cai et al. [2] (2021)	IEMOCAP	Accuracy: Unimodal: 58.97%
Wang and Han [52] (2021)	Proprietary	Accuracy: 89.89%
Graterol et al. [56] (2021)	Dataset E-c of SemEval-2018	Accuracy: 53.5%
Abdelhamid et al. [10] (2022)	RAVDESS, EMODB, SAVEE, IEMOCAP	Accuracy: RAVDESS: 99.47%, SAVEE: 99.50%, EMODB: 99.76%, IEMOCAP: 98.13%
Heredia et al. [57] (2022)	IEMOCAP	Accuracy: Audio and Text: 78%, Audio, Text and Face: 77.6%
Singh et al. [46] (2023)	RAVDESS, SAVEE and TESS	Accuracy: RAVDESS: 74.44%, SAVEE: 57.50% TESS: 99.81%, Combined (RAVDESS + SAVEE + TESS): 90.19%
Mishra et al. [47] (2023)	RAVDESS, EMO-DB and SAVEE	Accuracy: RAVDESS: 77.54% with combination of MFCCmean, MFCCAE and MFCCSE, EMO-DB: 87.48% with combination MFCCmean and MFCCSE, SAVEE: 79.64% with combination MFCCmean and MFCCSE.
Sha et al. [51] (2024)	IEMOCAP	Accuracy: WA: 75.31%, UA: 75.09%.
Lin et al. [60] (2024)	Version 1.8 of the MSP-Podcast corpus	Accuracy: Arousal: Temp-GRU: 57.22%, Valence: Temp-Trans: 15.86%, Dominance: Temp-CNN: 47.05%.
Deshmukh et al. [44] (2024)	EMO-DB and RAVDESS	Accuracy: EMO-DB: 95.76%, RAVDESS: 84.64%

**Table 4:** Error metrics for the validation set (first model).

Emotions	Accuracy	Recall	F1-Score	Support
Disgust	0.85	0.80	0.82	822
Anger	0.96	0.90	0.93	822
Happiness	0.92	0.87	0.90	822
Fear	0.79	0.87	0.83	822
Neutral	0.88	0.90	0.89	822
Surprise	0.95	0.96	0.95	822
Sadness	0.85	0.88	0.86	822
WA	0.89	0.88	0.88	5754

**Table 5:** Error metrics for the test set (first model).

Emotions	Accuracy	Recall	F1-Score	Support
Disgust	0.85	0.83	0.84	822
Anger	0.93	0.93	0.93	822
Happiness	0.92	0.87	0.90	822
Fear	0.82	0.87	0.84	822
Neutral	0.90	0.92	0.91	822
Surprise	0.95	0.96	0.95	822
Sadness	0.89	0.86	0.87	822
WA	0.89	0.89	0.89	5754

**Table 6:** Error metrics for the validation set (second model).

Emotions	Accuracy	Recall	F1-Score	Support
Disgust	0.90	0.89	0.90	822
Anger	0.88	0.89	0.89	822
Happiness	0.91	0.83	0.87	822
Fear	0.85	0.92	0.88	822
Neutral	0.87	0.89	0.88	822
Surprise	0.94	0.92	0.93	822
Sadness	0.87	0.86	0.87	822
WA	0.89	0.89	0.89	5754

**Table 7:** Error metrics for the test set (second model).

Emotions	Accuracy	Recall	F1-Score	Support
Disgust	0.92	0.89	0.91	822
Anger	0.91	0.89	0.90	822
Happiness	0.91	0.86	0.88	822
Fear	0.85	0.94	0.89	822
Neutral	0.88	0.90	0.89	822
Surprise	0.94	0.93	0.94	822
Sadness	0.87	0.85	0.86	822
WA	0.90	0.90	0.90	5754

**Table 8:** Error metrics for the validation set (third model).

Emotions	Accuracy	Recall	F1-Score	Support
Disgust	0.94	0.98	0.96	822
Anger	0.98	0.97	0.98	822
Happiness	0.97	0.95	0.96	822
Fear	0.97	0.95	0.96	822
Neutral	0.97	0.94	0.96	822
Surprise	0.96	0.98	0.97	822
Sadness	0.96	0.97	0.96	822
WA	0.96	0.96	0.96	5754

**Table 9:** Error metrics for the test set (third model).

Emotions	Accuracy	Recall	F1-Score	Support
Disgust	0.98	0.96	0.97	822
Anger	0.97	0.97	0.97	822
Happiness	0.97	0.96	0.97	822
Fear	0.96	0.98	0.97	822
Neutral	0.97	0.96	0.96	822
Surprise	0.97	0.98	0.98	822
Sadness	0.97	0.96	0.97	822
WA	0.97	0.97	0.97	5754

**Table 10:** Databases for neural network testing (Model 3: feature fusion).

Database	Emotions	Language	Category
EMOVO—Costantini et al. [69]	Neutral, Happiness, Sadness, Anger, Fear, Surprise, Disgust	Italian	Simulated
MESD—Duville et al. [70]	Neutral, Happiness, Sadness, Anger, Fear, Disgust	Spanish	Simulated

**Table 11:** Error metrics for the validation set (EMOVO).

Emotions	Accuracy	Recall	F1-Score	Support
Disgust	0.91	0.91	0.91	34
Anger	0.94	0.97	0.96	34
Happiness	0.92	0.71	0.80	34
Fear	0.94	0.88	0.91	34
Neutral	1.00	0.94	0.97	34
Surprise	0.72	0.97	0.82	34
Sadness	0.97	0.94	0.96	34
WA	0.91	0.90	0.90	238

**Table 12:** Error metrics for the test set (EMOVO).

Emotions	Accuracy	Recall	F1-Score	Support
Disgust	1.00	0.97	0.99	34
Anger	1.00	1.00	1.00	34
Happiness	1.00	0.88	0.94	34
Fear	0.89	0.91	0.90	34
Neutral	1.00	0.94	0.97	34
Surprise	0.82	0.97	0.89	34
Sadness	0.91	0.91	0.91	34
WA	0.95	0.94	0.94	238

**Table 13:** Error metrics for the validation set (MESD).

Emotions	Accuracy	Recall	F1-Score	Support
Disgust	1.00	0.92	0.96	79
Anger	0.95	0.99	0.97	75
Happiness	0.99	0.99	0.99	79
Fear	0.97	0.99	0.98	79
Neutral	0.99	1.00	0.99	75
Surprise	0.99	1.00	0.99	79
Sadness	0.99	1.00	0.99	79
WA	0.98	0.98	0.98	466

**Table 14:** Error metrics for the test set (MESD).

Emotions	Accuracy	Recall	F1-Score	Support
Disgust	0.95	0.95	0.95	76
Anger	0.96	0.97	0.97	76
Happiness	0.96	0.99	0.97	76
Fear	0.99	0.96	0.97	76
Neutral	0.96	0.95	0.95	76
Sadness	0.96	0.96	0.96	76
WA	0.96	0.96	0.96	456

## Data Availability

Data are contained within the article.
